# Stable Pseudohyphal Growth in Budding Yeast Induced by Synergism between Septin Defects and Altered MAP-kinase Signaling

**DOI:** 10.1371/journal.pgen.1005684

**Published:** 2015-12-07

**Authors:** Junwon Kim, Mark D. Rose

**Affiliations:** Department of Molecular Biology, Princeton University, Princeton, New Jersey, United States of America; SUNY-Buffalo, UNITED STATES

## Abstract

Upon nutrient limitation, budding yeasts like *Saccharomyces cerevisiae* can be induced to adopt alternate filament-like growth patterns called diploid pseudohyphal or invasive haploid growth. Here, we report a novel constitutive pseudohyphal growth state, sharing some characteristics with classic forms of filamentous growth, but differing in crucial aspects of morphology, growth conditions and genetic regulation. The constitutive pseudohyphal state is observed in *fus3* mutants containing various septin assembly defects, which we refer to as sadF growth (septin assembly defect induced filamentation) to distinguish it from classic filamentation pathways. Similar to other filamentous states, sadF cultures comprise aggregated chains of highly elongated cells. Unlike the classic pathways, sadF growth occurs in liquid rich media, requiring neither starvation nor the key pseudohyphal proteins, Flo8p and Flo11p. Moreover sadF growth occurs in haploid strains of S288C genetic background, which normally cannot undergo pseudohyphal growth. The sadF cells undergo highly polarized bud growth during prolonged G2 delays dependent on Swe1p. They contain septin structures distinct from classical pseudo-hyphae and FM4-64 labeling at actively growing tips similar to the Spitzenkörper observed in true hyphal growth. The sadF growth state is induced by synergism between Kss1p-dependent signaling and septin assembly defects; mild disruption of mitotic septins activates Kss1p-dependent gene expression, which exacerbates the septin defects, leading to hyper-activation of Kss1p. Unlike classical pseudo-hyphal growth, sadF signaling requires Ste5, Ste4 and Ste18, the scaffold protein and G-protein β and γ subunits from the pheromone response pathway, respectively. A *swe1* mutation largely abolished signaling, breaking the positive feedback that leads to amplification of sadF signaling. Taken together, our findings show that budding yeast can access a stable constitutive pseudohyphal growth state with very few genetic and regulatory changes.

## Author Summary

Many pathogenic fungi alternate between unicellular and multicellular filamentous forms, which is often critical for host-cell attachment, tissue invasion, and virulence. Certain strains of the nonpathogenic budding yeast *Saccharomyces cerevisiae* are also capable of forming invasive pseudohyphal filaments in nutrient poor conditions, which has served as a model system for the study of filamentous fungal pathogens. Here, we show that the most commonly used laboratory strain, S288c, previously known as being non-filamentous, can adopt a permanent stable pseudohyphal growth phase even under rich growth conditions. Although some features are shared, the degree of filamentation, genetic requirements, cell cycle, and mechanism of regulation are distinct from the previously described forms of filamentous growth. Stable pseudohyphal growth arises as a result of only two mutations, neither of which causes pseudohyphal growth on their own. One mutation causes subtle defects in the mechanism of cell separation (septation), which activate intracellular signaling pathways that slow cell division and promote filamentation. Normally this pathway is kept in check by a related signaling protein. However, when the inhibitor is also defective, activation of the filamentation signaling pathway exacerbates the septation defects, which causes a synergistic hyper-activation of pseudohyphal growth. These findings expand our understanding of fungal pathogenesis mechanisms at the molecular level.

## Introduction

Many fungal pathogens undergo a developmental transition from unicellular to multicellular filamentous forms that are important for the invasion of host tissue and virulence [1]. Certain strains of nonpathogenic *Saccharomyces cerevisiae* are also capable of developing filament-like growth under starvation conditions, which is thought to serve as a foraging mechanism. For example, diploid yeast cells starved for nitrogen exhibit pseudohyphal growth on solid agar medium [2]. Pseudohyphae consist of invasive filaments comprising chains of elongated cells that remain physically connected after cytokinesis, divide in a unipolar manner, and have an altered cell cycle to a prolonged budded period [2,3]. Haploid yeast undergoes a similar morphological transition called “haploid invasive growth” in response to glucose depletion. Under these conditions, haploid cells penetrate the agar, but do not become as elongated as cells in diploid pseudo-hyphal cells and do not form extensive filaments on the agar surface [4].

The regulation of the known patterns of dimorphic growth is complex, but requires at least two major signaling pathways, the filamentation mitogen activated protein kinase (fMAPK) and the nutrient-sensing cyclic AMP-protein kinase A (cAMP/PKA) pathway (reviewed by [5]). Both pathways coordinately upregulate *FLO11*, a cell surface flocculin; *flo11* mutant cells fail to form chains or invade agar in both haploid and diploid yeast.

The fMAPK pathway includes several protein kinases, Ste20p, Ste11p, Ste7p, and the MAPK Kss1p, which act sequentially to activate the transcription factor Ste12p/Tec1p heterodimer and regulate expression of genes responsible for filamentous growth [5]. Several elements of the fMAPK cascade are also essential for the pheromone response pathway required for mating. However, activation of the MAPK for mating, Fus3p, blocks the filamentation program by down-regulating Kss1p and Tec1p [6,7]. Signal specificity is also provided by the Ste5p scaffold protein, which transduces pheromone signaling after recruitment to the G-protein β/γ dimer (Ste4p/Ste18p) at the plasma membrane. Ste5p is absolutely required for activating Fus3p [8], but is not required for the fMAPK pathway. Although upstream signaling events for the fMAPK pathway are not well understood, Ras2p GTPase and the plasma membrane-linked receptors, Msb2p and Sho1p have been reported to be implicated in fMAPK activation, along with Cdc42p, the rho GTPase that activates Ste20p [5].

Ras2p activates a second signal transduction pathway by stimulating adenylate cyclase and cAMP production [9]. Increased cAMP levels activates the Tpk2p catalytic subunits of PKA, which phosphorylates and displaces the repressor Sf1lp, activating the key filamentous transcription factor Flo8p [5]. Many commonly used laboratory strains, including S288C, the reference strain for the yeast genome project, carry a *flo8* mutation and are unable to form pseudo-hyphal filaments or undergo haploid invasive growth [10]. A G-protein-coupled receptor (GPCR) called Gpr1p also appears to act upstream of adenylate cyclase, dependent on Gpa2p, a G-protein α subunit. Phospholipase Plc1p modulates the interaction of Gpa2p with Gpr1p [5].

Less clear are the cell biological mechanisms governing the morphological change to filamentous growth, as distinct from budding growth. It seems likely that at least three conditions must be met for cells to form filaments. First, individual cells must maintain a polarized pattern of growth, rather than the isotropic growth observed in budding cells. Second, because filamentous cells grow to much larger volumes than budding cells, they must alter the normal coupling between growth and mitosis. Finally, for cells to stay together as filaments, they must suppress the completion of septation to form long chains.

Cytokinesis, septation, and polarized growth are governed in part by a conserved family of proteins called septins. The yeast septins (Cdc3p, Cdc10, Cdc11p, Cdc12p and Shs1p) polymerize into membrane-associated filaments that form an ordered ring structure at the future budding site. During mitosis the septin ring expands into an hourglass-shaped collar that serves as a diffusion barrier and as a scaffold to direct a variety of cellular processes, including morphogenesis and bud site selection (reviewed by [11]). The formation of higher-ordered septin structures requires several post-translational modifications (reviewed by [12]). The protein kinases Elm1p, Gin4p and Cla4p promote proper septin assembly. Both Gin4p and Cla4p phosphorylate the septins and regulate the dynamics and organization of septin filaments [13,14]. Cla4p is also required for Gin4p activation [15] and recruitment to the bud neck and septins [13,16]. Elm1p directly phosphorylates Gin4p [17] and is required for mitotic hyper-phosphorylation of Cla4p [18]. In addition, genetic data suggests that each kinase functions in parallel to regulate septin behavior [16,19]. Proper organization of the septins at the bud-neck is critical for coupling morphogenesis and cell cycle progression (reviewed by [20]). Defects in septin assembly lead to accumulation of Swe1p, an inhibitory kinase of mitotic CDK. As a result, septin-defective cells, including *elm1*, *gin4*, and *cla4* mutants, fail to switch from apical growth to isotropic growth, leading to formation of elongated buds.

We have identified a novel constitutive pseudohyphal growth phase of yeast distinct from the previously described pseudohyphal and haploid invasive growth patterns. Although some features are shared, many others are different, including the degree of filamentation, genetic requirements, and mechanism of regulation. Constitutive pseudohyphal growth arises from synergism between septin assembly defects and loss of Fus3p. The ease by which budding yeast can adopt a stable pseudohyphal growth state is consistent with recent findings that the transition from unicellular to filamentous growth requires relatively few regulatory changes.

## Results

### Identification and characterization of constitutive pseudo-hyphal growth

**Fig 1:**
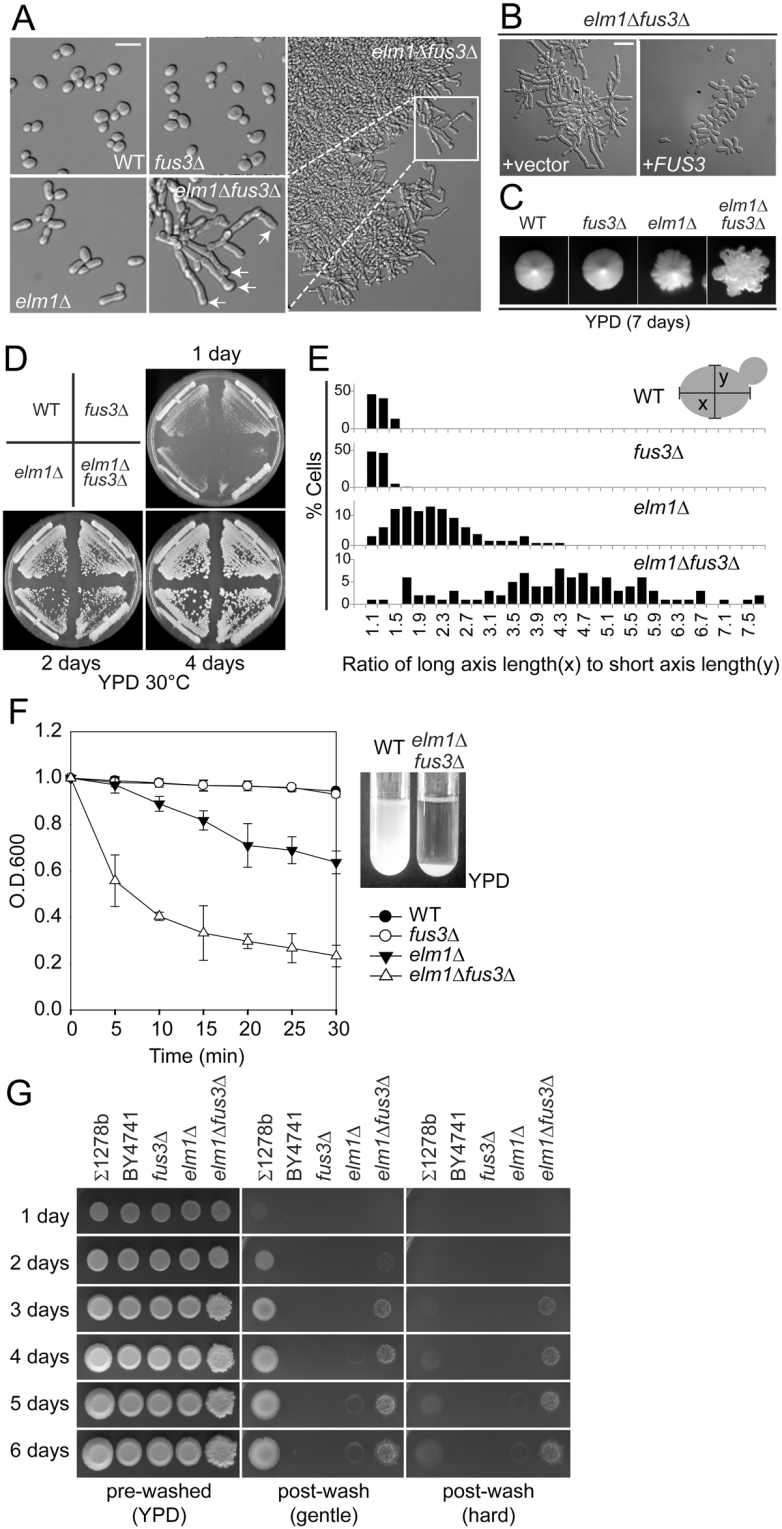
Filamentous growth of *elm1*Δ *fus3*Δ cells. All of indicated strains (MY8092, 12158, 12886 and 12948) were BY4741-derivative haploids and grown to exponential phase in liquid YPD at 30°C unless stated otherwise. (A) Arrows indicate the buds that emerge from the pole opposite previous division site. (B) *elm1*Δ *fus3*Δ (MY12948) harboring empty vector (MR1865) or *pCEN-FUS3* (MR5048) were grown in liquid synthetic complete (SC) medium containing 2% glucose. (C) Cells were streaked for single cells on YPD plate at 30°C for 7 days, and representatives in a zone of low colony density were photographed. (D) Indicated cells (MY8092, 12158, 12886 and 12948) were streaked on YPD solid medium and incubated at 30°C. (E) The ratio of long axis (x) to short axis length (y) was expressed in a histogram. For example, 1.1 and 1.3 at the x-axis indicate the range from 1.00 to 1.19 and 1.20 to 1.39, respectively. (F) Flocculation assay was performed as described in Materials and methods. Data are representative of three independent experiments and expressed relative to WT. (G) Cells were spotted on YPD plate and grown at 30°C for 6 days. Haploid Σ1278b (MY13465) was used as a positive control of invasive growth. (A and B) Bar, 10 μm.

Strain BY4741, the reference strain for the yeast genome, is a haploid derived from the filamentation-deficient S288C background. In the course of routine strain construction, we observed that *elm1*Δ *fus3*Δ double mutants exhibited unusual colony and cell morphologies (Fig 1). *ELM1* encodes a protein kinase that regulates septins and cytokinesis. The *elm1*Δ caused elongated buds and slightly rough colony morphology (Fig 1A and 1C). Fus3p is a pheromone-activated MAP kinase and functions during mating [21]. Although *fus3*Δ had no obvious effect on the cell or colony morphology in the wild-type (WT) background, combination with *elm1*Δ led to dramatic morphological changes (Fig 1A). The *elm1*Δ *fus3*Δ cells grew as large aggregates in liquid YPD medium, forming extensive filaments comprising chains of highly elongated cells. The abnormal morphology was completely reversed by a *CEN* plasmid carrying *FUS3* (Fig 1B).

Colony morphology can be influenced both by growth conditions and by cellular morphology. Colonies of *fus3*Δ displayed a smooth circular outline with a lustrous surface, identical to WT (Fig 1C). The *elm1*Δ colonies exhibited a slightly notched outline (Fig 1C). In contrast, *elm1*Δ *fus3*Δ colonies were highly ruffled and rough-edged, with filaments extending beyond the perimeter of the colony (Fig 1C). Colonies of *elm1*Δ *fus3*Δ were comparable in size to WT, indicating that they grow with a similar rate as WT (Fig 1D).

The morphology of the *elm1*Δ *fus3*Δ cells resembled diploid pseudohyphal filaments; both cells are highly polarized and form extensive surface-spread filaments. However, the *elm1*Δ *fus3*Δ filaments are clearly distinct from pseudohyphal growth, which is restricted to diploid cells, occurs only on solid medium deficient in nitrogen, and requires *FLO8* [2]. In contrast, the *elm1*Δ *fus3*Δ filaments formed from haploid cells, carrying a *flo8* mutation, and grew stably in rich liquid media (YPD). Given these differences, and because of significant differences in regulation described later, we refer to the morphology observed in the *elm1*Δ *fus3*Δ mutant as “septin assembly defect induced filamentous” growth (sadF).

One major characteristic of sadF growth is the extreme elongation of the cells. In WT cells the ratio of the long axis to the short axis was 1.22 ±0.15 (n = 112), which was not affected by the *fus3*Δ (1.21 ±0.13, n = 106) (Fig 1E). The *elm1*Δ had a ratio of 2.15 ±0.67 (n = 132), consistent with their slight elongation. In contrast, *elm1*Δ *fus3*Δ was almost 4 times longer than WT (4.31 ±1.48, n = 101) (Fig 1E). Thus, the *fus3*Δ mutation greatly enhanced polarized cell growth of *elm1*Δ.

The *elm1*Δ *fus3*Δ strain grew in large multicellular clumps (Fig 1A), suggesting a high level of cell-cell adhesion. To assess the level of aggregation, we used a spectrophotometric assay to measure the rate at which cells settled out of liquid culture. Both WT and *fus3*Δ cultures showed very little decrease in optical density (Fig 1F) and the *elm1*Δ showed a slight decrease. In contrast, *elm1*Δ *fus3*Δ cells rapidly settled out of suspension, with a half-time less than 5 minutes; almost all settled by 15 minutes. Thus the *fus3*Δ mutation enhanced greatly cell-cell adhesion in *elm1*Δ.

Next, we examined if *elm1*Δ *fus3*Δ cells are able to penetrate solid growth media as do pseudohyphal diploids and invasive haploids. Non-adherent cells were easily washed off with a stream of water, after which two classes of cells remained: invasive cells and adherent non-invasive cells (Fig 1G, gentle post-wash). Adherent non-invasive cells can be removed by vigorous rubbing of the agar surface under running water (Fig 1G, hard post-wash). Haploid Σ1278b, a standard background for filamentous growth, began to penetrate the solid medium after 3 days, as a result of glucose depletion. In contrast, both WT haploid BY4741 and *fus3*Δ failed to invade even after prolonged growth (Fig 1G, hard post-wash). The *elm1*Δ strain exhibited very weak invasiveness (Fig 1G). Remarkably, agar penetration by *elm1*Δ was enhanced strongly by *fus3*Δ. Although *elm1*Δ *fus3*Δ surface cells were easily removed by gentle washing, showing that the strain is less adherent than Σ1278b (Fig 1G, gentle post-wash), a significant portion invaded the agar (Fig 1F, hard post-wash). Agar penetration began to be visible after 2 days and increased over time. These data suggest that *elm1*Δ *fus3*Δ pseudohyphal growth is more invasive, but less adherent, than haploid invasive growth.

### Highly polarization and altered budding pattern in sadF growth

**Fig 2:**
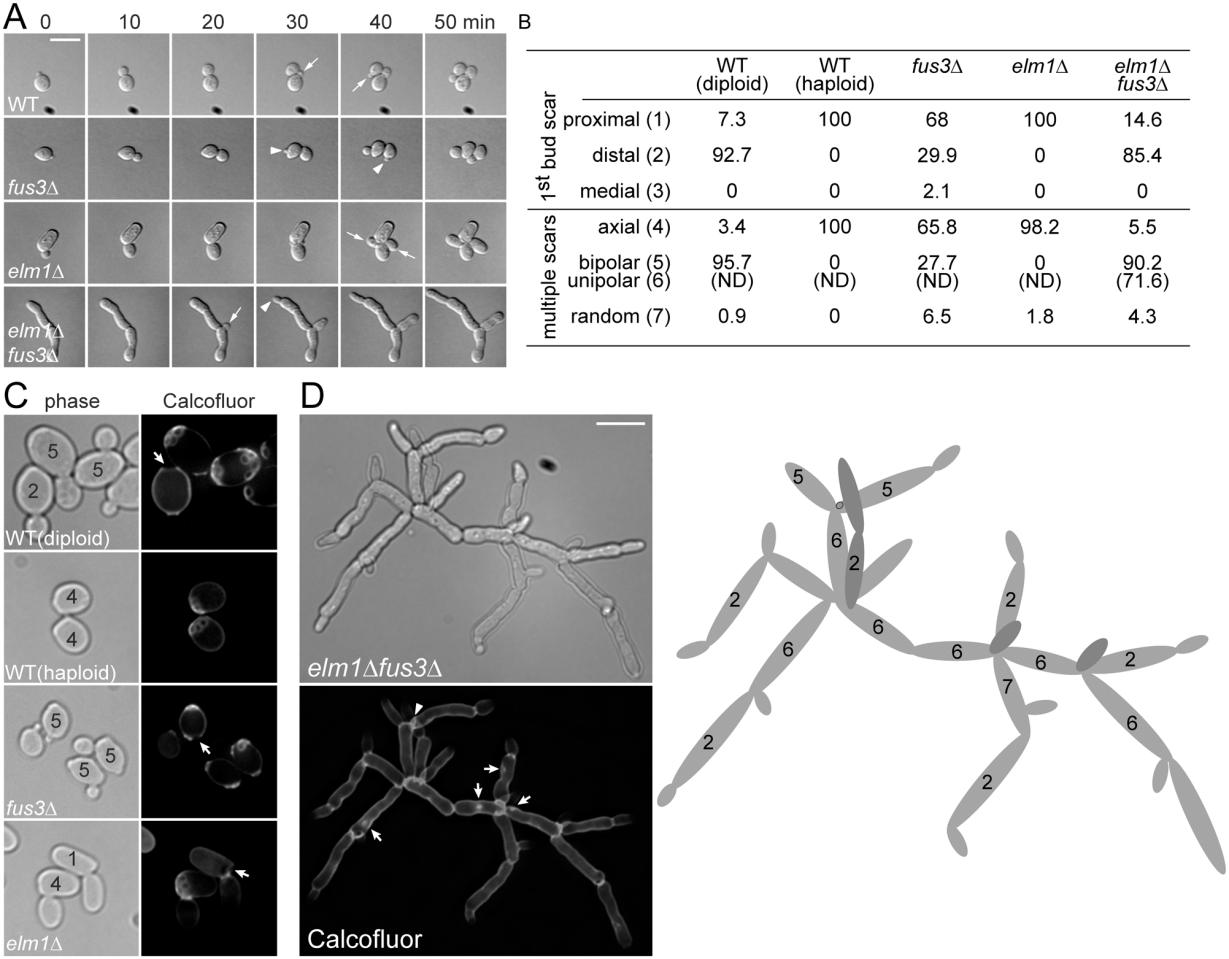
Budding pattern of *elm1*Δ *fus3*Δ cells. All of indicated strains (MY8092, 12158, 12886 and 12948) were BY4741-derivative haploids and grown to exponential phase in liquid YPD at 30°C. S288C-derivative diploid (MY13411) was used as a positive control of bipolar budding. (A) Budding pattern was examined by time lapse microscopy as described in Materials and methods. Representative images are shown at 10-min interval. Arrows and arrow heads indicate the daughters that emerge adjacent or opposite to the preceding division site, respectively. (B) Cells were classified by number and distribution of bud scars. For each sample, more than 200 cells were counted and the score was expressed as a percentage. N.D, not determined. (C and D) Cells were stained with 0.1% calcofluor. The classified budding patterns are denoted as numbers that correspond to that of (B). (C) Arrows indicate birth scar. (D) Representative cluster of *elm1*Δ *fus3*Δ cells is shown with an interpretative drawing of the budding pattern (right). Arrow head (left) and black ring (drawing) indicate a bud scar. Arrows indicate abnormal chitin accumulations. (A, C, and D) Bar, 10 μm.

The reiteration of the axial budding pattern in haploids produces tight clusters of cells that form mounds on the surface of solid media ([22] and Fig 1C). In contrast, *elm1*Δ *fus3*Δ cells budded from the pole opposite the previous division site (distal pole, Fig 1A, arrows) and formed mats of branching chains of cells extending beyond the colony margin (Fig 1C). To characterize the *elm1*Δ *fus3*Δ growth pattern in detail, we used time lapse microscopy. Cells were sonicated lightly and applied to YPD agar pads. Because most *elm1*Δ *fus3*Δ cells are in large clumps, cells remaining in suspension were observed. Bud site selection was determined for all buds that emerged after the initial observation; none of the cells present at time zero was scored, as the previous division site could not always be determined. In all of 14 WT and 12 *elm1*Δ cells, the new buds emerged adjacent to the first mother/daughter bud site (proximal pole; Fig 2A). In contrast, in 11 *elm1*Δ *fus3*Δ cells the new buds emerged at the distal pole; in 10 cells they emerged at the proximal pole (Fig 2A). Surprisingly, even with *fus3*Δ, budding was not always axial (Fig 2A); 5 of 14 buds formed at the distal pole and 9 at the proximal pole (Fig 2A). These results show that Fus3p is required for the normal axial budding pattern and its loss induces the polar budding pattern that is critical for growth and extension of a filament [2].

To further quantify the budding pattern, cells were stained with calcofluor white, which binds to chitin in the cell wall, most strongly at the bud scars (Fig 2B–2D). Cells with 1 bud scar were divided into three classes based on the position of the bud scar relative to the birth scar: (1) proximal, (2) distal, and (3) medial. Cells with 2 or more bud scars were divided into three classes: (4) axial, if all bud scars are adjacent at one end of the cell, (5) bipolar, if all bud scars are positioned at the distal pole or distributed between the proximal and distal pole, and (7) random, if one or more bud scars is positioned in the midsection. WT haploids always had the first bud scar proximal to their birth scar and multiple bud scars were clustered in a chain at one end of the cells. The *elm1*Δ haploids behaved like WT (Fig 2B and 2C). Consistent with the time-lapse microscopy, only 66% of the *fus3*Δ cells exhibited the axial budding pattern; in about 30% of cells the first bud was opposite the birth scar and 28% of cells with multiple bud scars showed bipolar budding (Fig 2B and 2C). As expected, WT diploids always budded in the bipolar mode (Fig 2B and 2C).

**Fig 3:**
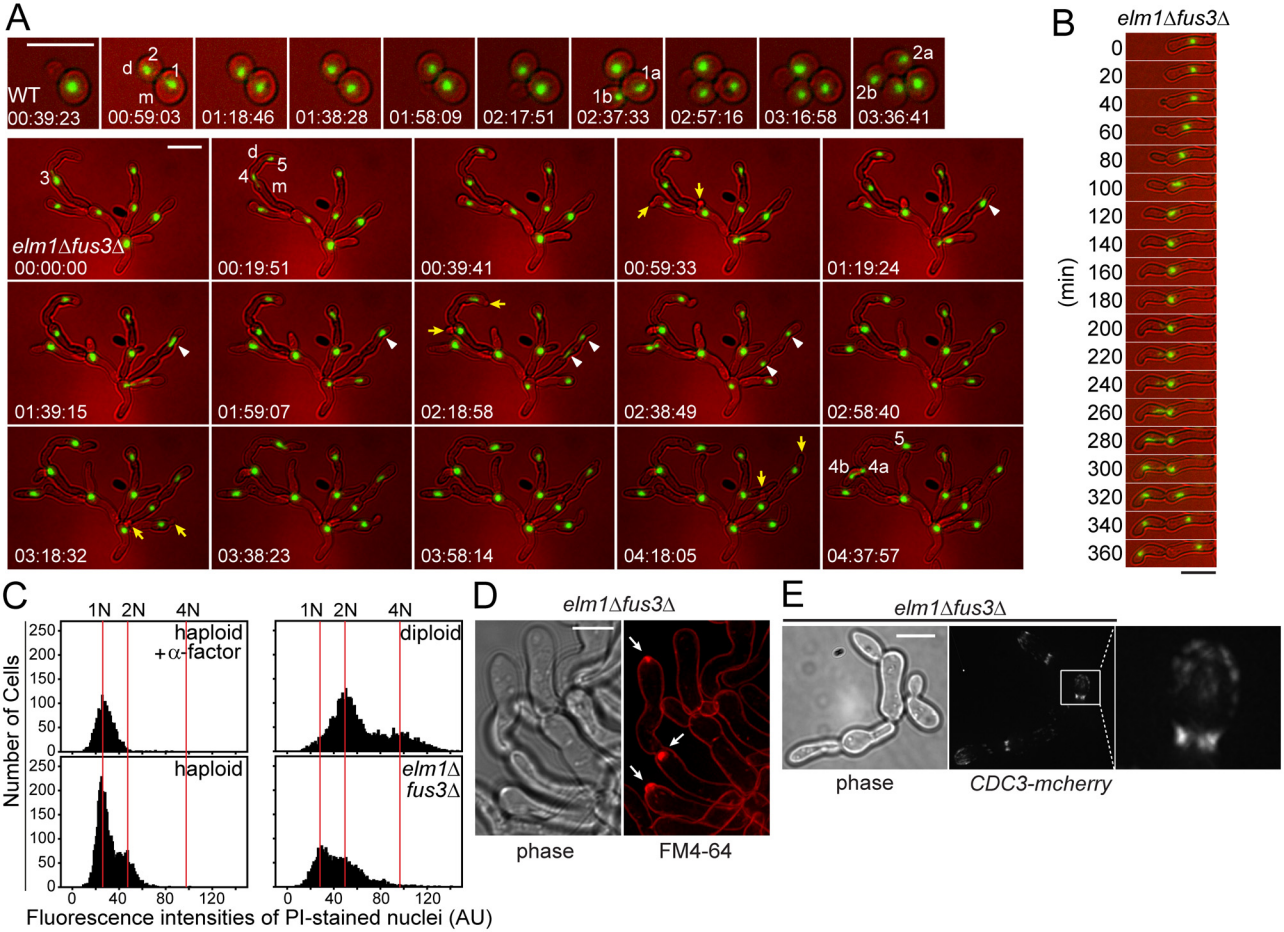
Cell biological characteristics of *elm1*Δ *fus3*Δ cells. All cells were grown to exponential phase in liquid YPD at 30°C. (A and B) The nuclei of indicated strains carrying *HTB1-GFP* (MY13817 and 13820) were examined by time lapse microscopy as described in Materials and methods. Representative images are shown at 20-min interval. (A) Sequential nuclear division is indicated by the number. For example, the nucleus #1 that had been just divided was segregated into two nuclei (1a and 1b) during the next round of cell cycle. Arrows indicate the daughter cells that emerge synchronously. Arrow heads show the nuclear division in mother cells. m, mother. d, daughter. (C) Indicated cells (MY8092, 12948 and MY13411) were stained with propidium iodide and their intensities were expressed in a histogram as described in Materials and methods. 10 μg/ml α-factor was treated to arrest cells in G1 with 1N DNA content. (D) *elm1*Δ *fus3*Δ (MY12948) were stained with FM4-64 to visualize the plasma membrane. Arrows indicate an intensive fluorescent accumulation, Spitzenkörper-like structure. Bar, 5 μm. (E) Indicated cells (MY13161 and 13264) were examined to visualize septin, Cdc3-mCherry. (A, B and E) Bar, 10 μm.

The *elm1*Δ *fus3*Δ filamentous aggregates could not be dispersed to single cells even by extensive sonication, suggesting that the cells remained strongly attached. Staining with calcofluor showed greatly enhanced staining over most of the cell wall except for the distal tips (Fig 2D), unlike WT in which staining was confined to bud scars and septa (Fig 2C). Although staining was observed at abnormal sites (arrows), most cells showed intense staining at the junction between cells, suggesting the presence of a complete septum (Fig 2D). Staining of the plasma membrane with the fluorescent styryl dye FM4-64 showed most bud necks had plasma membrane except where buds were growing at the end of cell chains (Fig 3E). These data suggest that although septum formation may be abnormal, the elongated cells do eventually complete cytokinesis. We conclude that chain formation is largely due to decreased abscission.

Analysis of the budding pattern of the smaller *elm1*Δ *fus3*Δ clusters supported the hypothesis that they are defective for cell separation. A representative cell is shown in Fig 2D with an interpretative drawing of the budding pattern. In this cluster, only one clear bud scar (arrow head and black ring in the carton) is visible, suggesting that very few cells have separated from the cell mass. Nevertheless, the branching pattern allows one to deduce the budding history. Most new buds (85%) were positioned at the distal pole and the bipolar budding pattern increased to 90%, compared to 28% of *fus3*Δ (Fig 2B–2D). Only 5.5% exhibited axial budding (Fig 2B and 2D). Importantly, among cells classed as “bipolar”, 72% exhibited a more polarized unipolar budding pattern (Fig 2B and 2D), a characteristic of diploid pseudohyphal growth in which new buds form only at the distal poles of both the mother and the daughter cells. Taken together, our data show that sadF growth is due to extreme cell elongation, coupled with to unipolar budding, resulting in extensive growth beyond the colony margin as seen in Fig 1C.

### Cell biological characteristics of sadF growth

The filamentous nature of *elm1*Δ *fus3*Δ raises the question of how nuclear division is coupled to cell division, and whether the cells are multi-nucleate or uninucleate. To monitor cell cycle progression, we visualized the nucleus using GFP-tagged histone, Htb1p. WT displayed the typical asymmetric cell division in which a bud emerged on the mother prior to bud emergence on the daughter. The interval between two sequential nuclear divisions defines the cell cycle time. For WT (Fig 3A), the nucleus #1 in the mother (m) divided after about 1h 30min, while the nucleus #2 in the daughter (d) divided after about 2h 30min. Similar times were observed for other cells (1h 56 min ± 14, for the mother, n = 14; 3h 9 min ± 35, for daughters, n = 15). In contrast, *elm1*Δ *fus3*Δ cells commonly showed simultaneous bud emergence on both the mother and daughter (Fig 3A, arrows). The cell cycle was significantly longer and more variable, compared to WT. For example, after nucleus #3 divided into two nuclei, it took 4h 20min for nucleus #4 in the mother (m) to divide while nucleus #5 in the daughter (d) remained undivided over the time course (5h 30min). In 8 of 14 cells, the time between nuclear divisions was almost 3 times longer than WT (5h 33min ±48); in 6 cells the nucleus remained undivided by 8h.

In most *elm1*Δ *fus3*Δ cells, the nucleus was located near the bud neck just prior to division (Fig 3B), as in WT. When the bud emerged, a centrally located nucleus migrated close to the bud neck. The bud continued to grow apically until mitosis occurred and one daughter nucleus migrated into the daughter cell. Often, the nucleus retained its aberrant position, leading to division in the mother or daughter cell (Fig 3A, arrow heads). In these cases, one of the nuclei always moved into the mother or daughter cell, followed rapidly by formation of a septum. Thus, each segment in the filament contained a single nucleus. After nuclear separation, the nuclei remained in separate cell compartments, supporting the suggestion that complete septa have formed.

The slow nuclear cell division cycle might arise if DNA replication occurred without mitosis. We therefore examined the DNA content by quantitative fluorescence microscopy (fluorescence-activated cell sorting was not feasible with the filamentous cells). WT haploids arrested with α-factor yielded a single peak, which corresponds to 1N DNA content (Fig 3C). An asynchronously growing WT haploid strain was a mixture of 1N and 2N cells, with most cells 1N. Note that post-metaphase nuclei are 1N in this experiment. Similarly, an isogenic diploid yielded two unequal peaks corresponding to 2N and 4N (Fig 3C). The *elm1*Δ *fus3*Δ cells also had two peaks, coincident with 1N and 2N, with no peak at 4N, showing that these cells are haploid. However, a significantly higher fraction of the cells had 2N DNA content, indicating that many are paused in G2, consistent with a delay in nuclear division after replication. In spite of the dramatically slowed nuclear division cycle, the growth rate of *elm1*Δ *fus3*Δ was comparable to WT at 30°C (Fig 1D). Therefore, the continued elongation of cells in G1 and the extended G2 phase compensate for the reduced rate of cell division.

FM4-64 binds to the plasma membrane bilayer and only enters the cell via endocytosis. Unexpectedly, FM4-64 staining revealed discrete bright spots of fluorescence at the tips of growing filamentous cells and nascent branch points (Fig 3D, arrows). These bright spots are reminiscent of Spitzenkörper, which can be transiently visualized with endocytic FM4-64 and act as an organizing center to concentrate vesicles to the highly polarized, actively growing tips of hyphae in filamentous fungi [23]. Thus, as for filamentous fungi, pseudohyphal growth might be associated with a Spitzenkörper-like structure, consistent with highly polarized cell growth.

One of fundamental differences between pseudo and true hyphae is the septin cytoskeleton [24]. The dimorphic fungal pathogen *C*. *albicans* grows as budding yeast, but also forms both pseudo and true hyphae. During *Candida* budding, the septin structure is similar to that of *Saccharomyces* budding. However, the localization and organization of the septins are distinct during hyphal growth (rings, bars, and caps [24]). In hyphal development a band of longitudinal septin bars and a cap of septin form at the base or tip of germ tube respectively. Because *elm1*Δ *fus3*Δ cells showed one characteristic of true hyphae, a Spitzenkörper-like structure, we examined the septin organization, using Cdc3-mCherry (Fig 3E). Strikingly, septins in the *elm1*Δ *fus3*Δ assembled rings composed of discrete and parallel septin bars at the bud neck, and were also found as a diffuse cap at the growing tip as observed during *Candida* hyphal growth. These results further indicate that the pseudohyphae of *elm1*Δ *fus3*Δ are distinct from classical pseudohyphae.

### Induction of pseudohyphal growth by disruption of mitotic septin assembly

**Fig 4:**
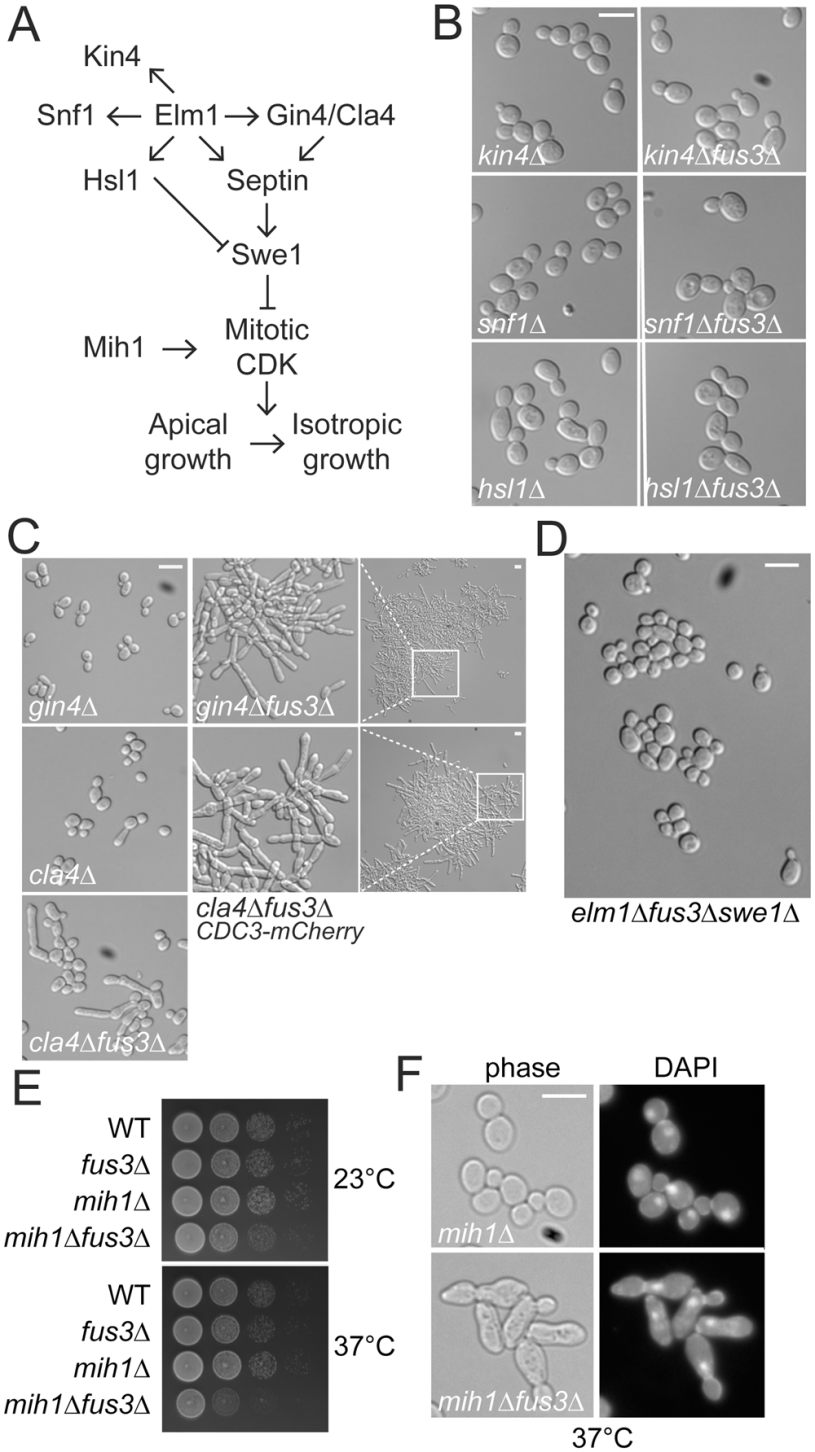
Genetic interaction of *fus3*Δ with septin associated genes. All of strains were BY474-derivative haploids and grown to exponential phase in liquid YPD at 30°C unless stated otherwise. (A) Schematic representation of Elm1p substrates and a morphogenesis checkpoint. For details, see Introduction. (B-D) Indicated strains (MY8092, 13826, 13827, 13829, 13830, 13832, 13833, 12156, 12871, 12886, 12941, 12944, 12990, 13125) was photographed. (E) Indicated cells (MY8092, 12886, 12960 and 12961) were serially diluted on YPD and incubated at either 23°C for 2 days or 37°C for 1 day. (F) MY12960 and 12961 cells grown to exponential phase at 23°C were transferred to 37°C for 12 hr. Cells were fixed with 3.7% formaldehyde and stained with DAPI. (B-D, and F) Bar, 10 μm.

The bud-neck protein kinase Elm1p has multiple substrates that regulate distinct cellular functions (Fig 4A). To determine which functions are associated with sadF pseudohyphal growth, we examined strains carrying mutations in known Elm1p substrates. Kin4p is a component of the spindle-position checkpoint [25]. Elm1p is also an upstream kinase for Snf1p, the yeast AMP-activated protein kinase (AMPK) [26]. Finally, Elm1p functions in a morphogenesis checkpoint pathway by phosphorylating Hsl1p, which coordinates bud growth and the G2/M transition by recruiting the Cdk-inhibitory kinase Swe1p to the bud neck for degradation [27]. Although deletions in each of these genes caused slight changes in the average axial ratio, in no case did the combination with *fus3*Δ lead to a significant increase in bud elongation (Fig 4B). Moreover, *fus3*Δ had no significant effect on the growth rate of *kin4*Δ, *snf1*Δ or *hsl1*Δ.

Elm1p contributes to proper formation of the septin cytoskeleton redundantly and/or partially through the protein kinases Gin4p and Cla4p (Fig 4A). To determine if disruption of septin organization is generally associated with pseudohyphal growth, we deleted *FUS3* in *gin4*Δ and *cla4*Δ strains. Each mutant displayed a slightly elongated cell phenotype, compared to WT (Fig 4C; *gin4*Δ, 1.46±0.29, n = 164; *cla4*Δ, 1.32±0.30, n = 139; WT, 1.22 ±0.15, n = 112). However a significant number of *gin4*Δ cells were hyper-elongated by loss of Fus3p (3.13±0.99, n = 118; Fig 4C) and formed large aggregates of cells that rapidly settled out of solution (S1A Fig). Like *elm1*Δ, the *fus3*Δ mutation caused enhanced agar penetration of the *gin4* mutant (S1B Fig). Similar results were observed with *cla4*Δ, but the combined effects with *fus3*Δ on polarized cell growth (1.91±1.11, n = 225), clumping and invasiveness were not as pronounced as for the *gin4*Δ mutants (Fig 4C and S1 Fig). However when the septin Cdc3p was tagged with mCherry, the *cla4*Δ *fus3*Δ cells displayed dramatic increases in cell elongation, cell-cell adhesion, and invasiveness (Fig 4C and S1 Fig). Although carboxy-terminally tagged Cdc3p-mCherry is thought to be fully functional, we observed slightly elongated buds, suggesting that it causes a slight septin defect. Taken together, the phenotypes of the *elm1*Δ *fus3*Δ, *gin4*Δ *fus3*Δ, and *cla4*Δ *fus3*Δ mutants suggest that mitotic septin defects are responsible for induction of pseudohyphal growth.

To further establish the relationship between Fus3p and septin perturbation in pseudohyphal growth, we examined the effect of *fus3*Δ on septin mutants. Four mutations (*cdc3-3*, *cdc10*Δ, *cdc12-6*, and *shs1*Δ) caused strong synthetic growth defects in combination with *fus3*Δ (S2A Fig). Two of the double mutants, *cdc3-3 fus3*Δ and *cdc12-6 fus3*Δ, displayed more severe morphologies with extremely elongated buds at the intermediate temperature of 30°C (S2B Fig). Taken together, these observations demonstrate that the loss of Fus3p exacerbates the effects of septin perturbation on cell morphology.

Perturbations of septin assembly lead to stabilization of Swe1p and a cell cycle delay at the G2/M transition, while buds undergo constitutive polarized growth [16]. A role for Swe1p in sadF pseudohyphal growth was demonstrated by finding that the *swe1*Δ completely suppressed the elongated cell morphology of *elm1*Δ *fus3*Δ cells (Fig 4D). The greatly enhanced polarization of *elm1*Δ *fus3*Δ, relative to *elm1*Δ, suggests that Swe1p may be further stabilized by loss of Fus3p. Mih1p promotes entry into mitosis by dephosphorylating the Swe1p-catalyzed inhibitory phosphorylation of Cdk1p [28]. If the loss of Fus3p affects the stability of Swe1p, then the *fus3*Δ *mih1*Δ double mutant should also show increased polarized bud growth and G2/M delay, relative to *mih1*Δ. Consistent with this hypothesis, *fus3*Δ caused increased bud elongation in *mih1*Δ and the double mutant exhibited defects in cell growth. These effects were more severe at elevated temperature (37°C), where most of cells were arrested with a single nucleus at the bud neck, indicative of G2 arrest (Fig 4E and 4F). However, because the *fus3*Δ *mih1*Δ mutant does not form chains of elongated cells, the effect of *elm1*Δ *fus3*Δ must entail more than Swe1p stabilization.

### Activation of Kss1p MAP kinase signaling by septin defects

**Fig 5:**
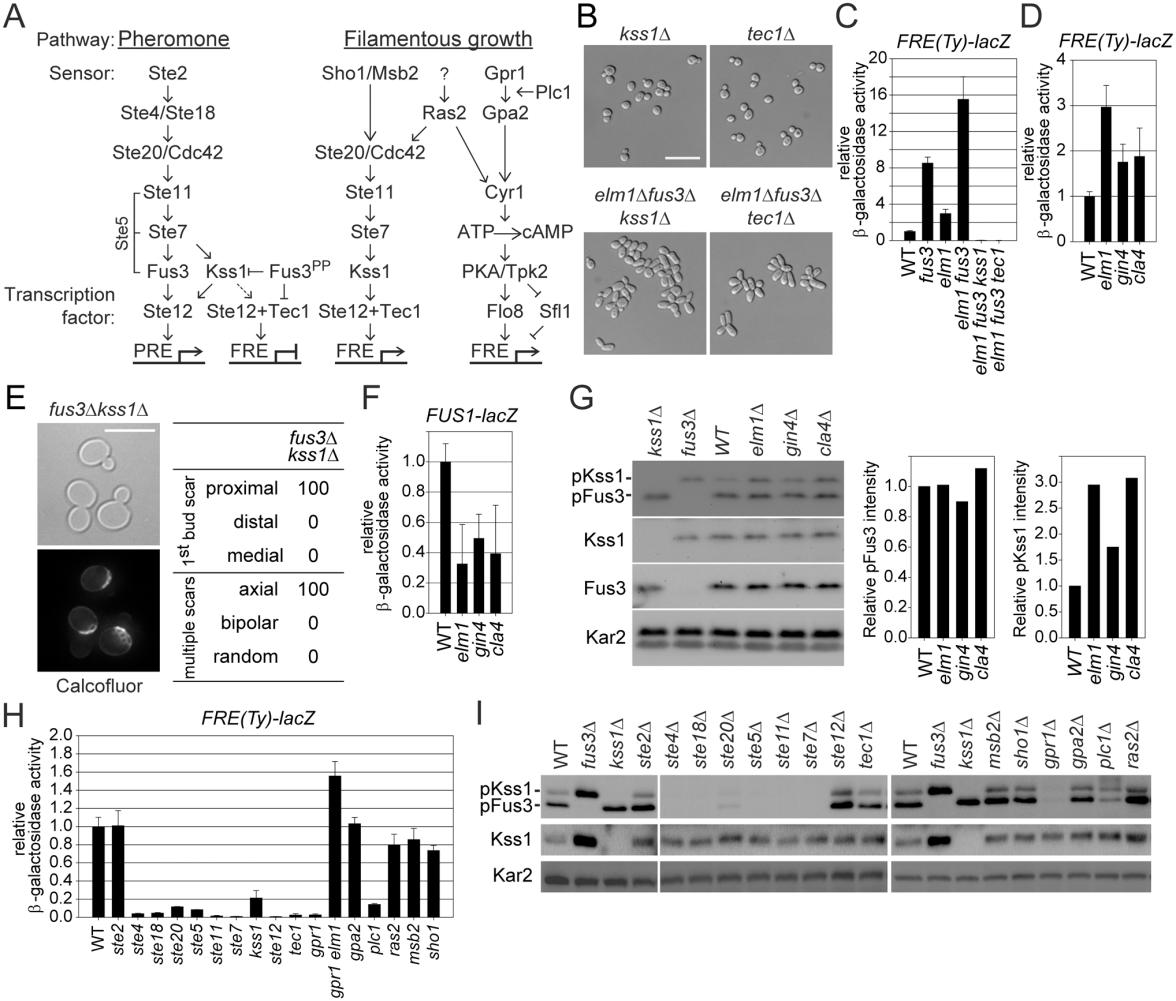
Upregulation of Kss1p-dependent signaling by *fus3*Δ mutation and septin assembly defects. All of strains were BY474-derivative haploids and grown to exponential phase in liquid YPD at 30°C unless stated otherwise. (A) Schematic representation of pheromone and filamentous growth pathway. For details, see the Introduction. (B) MY13049, 13122, 13144 and 13281 were photographed. (C and D) Indicated strains (MY8092, 12156, 12158, 12886, 12941, 12948, 13122, and 13281) harboring *FRE(Ty)-lacZ* (MR6857) were used. (E) The budding pattern of *fus3*Δ *kss1*Δ (MY13289) was analyzed as described in Fig 2B–2D. (F and G) MY8092, 12156, 12941 and 12948 strains were used. (F) Strains carry *FUS1-lacZ* (MR0727). (G) Intensities of phosphorylated Fus3p and Kss1p were normalized to total Fus3p and Kss1p and expressed relative to WT. (H) Indicated strains (MY8092, 13049, 13144, 13484, 13523, 13525, 14319, 14320, 14321, 14322, 14323, 14324, 14325, 14326, 14327, 14328, 14329 and 14330) harboring *FRE(Ty)-lacZ* (MR6857) were used. (I) Indicated strains (MY8092, 12886, 13049, 13144, 13523, 13525, 14319, 14320, 14321, 14322, 14323, 14324, 14325, 14326, 14327, 14328, 14329 and 14330) were used. (C, D, F, and H) Cells were grown to exponential phase in liquid SC containing 2% glucose. The activity of β-galactosidase was determined as described in Materials and Methods and expressed relative to wild type. Data are mean ± standard deviation from three independent experiments. (D and H) WT are identical to (C). (G and I) Active Fus3p and Kss1p were shown by phospho-p42/44 MAPK antibody. Total Fus3p, Kss1p and Kar2p were detected with anti-Fus3p, -Kss1p and -Kar2p respectively. (B and E) Bar, 10 μm.

Pheromone-activated Fus3p blocks classical pseudohyphal growth by down-regulating Kss1p and Tec1p (Fig 5A; [29]). Because deletion of either *KSS1* or *TEC1* also eliminated the sadF pseudohyphal phenotype of *elm1*Δ *fus3*Δ (Fig 5B), we monitored the expression of a Tec1p-dependent filamentation response element (FRE). Expression of a *FRE(Ty)-lacZ* reporter was synergistically elevated 15 fold in *elm1*Δ *fus3*Δ relative to WT (8-fold in *fus3*Δ, and 3-fold in *elm1*Δ), which was obliterated by either *kss1* or *tec1* mutation (Fig 5C). *FRE-lacZ* expression was also elevated in the other septin assembly mutants, *gin4*Δ and *cla4*Δ, although less than in *elm1*Δ (Fig 5D). These data show that septin disruption upregulates Kss1p-dependent signaling, contributing to sadF pseudohyphal growth. Moreover, *kss1*Δ restored the normal axial budding pattern to a *fus3*Δ mutant (Figs 2 and 5E), suggesting that Kss1p signaling regulates the normal haploid budding pattern.

How could Kss1p-dependent signaling be activated by septin assembly defects? Disruptions of mitotic septin organization and function could stimulate pheromone signaling, leading to increased activation of Kss1p in the absence of Fus3p inhibition. Expression of the mating-specific *FUS1*-*lacZ* reporter was activated >100-fold by pheromone. In contrast, the basal expression was reduced 2–3 fold in the *elm1*, *gin4*, or *cla4* mutants (Fig 5F). Because there is a basal level of Kss1p-dependent signaling, septin defects might reduce the kinase activity and/or level of Fus3p, resulting in the upregulation of Kss1p signaling. However, neither the protein level nor the phosphorylation of Fus3p was significantly affected by *elm1*Δ, *gin4*Δ, or *cla4*Δ (Fig 5G). In contrast, the level of phosphorylated Kss1p was significantly elevated in *elm1*Δ, *gin4*Δ, and *cla4*Δ cells. Taken together, we conclude that septin assembly defects trigger signaling through Kss1p, independent of pheromone signaling and Fus3p.

To identify upstream regulators of the constitutive activation of Kss1p, we screened candidate deletion mutations in BY4741 for their effects on *FRE-lacZ* expression during vegetative growth. Basal *FRE*-driven expression was greatly reduced in strains lacking elements shared by both the pheromone response and pseudohyphal pathways, including *ste20*, *ste11*, *ste7* and *ste12* (Fig 5A and 5H). Remarkably, *FRE*-driven expression was also abolished by loss of components specific to the pheromone response, including Ste4p (G_β_), Ste18p (G_γ_), and Ste5p (scaffold), but not by loss of the pheromone receptor, Ste2p. Ras2p and the plasma membrane-linked receptors Msb2p and Sho1p have been implicated in the activation of the fMAPK cascade during filamentous growth (Fig 5A). However, they are not required for basal signaling; null mutants showed ~80% WT expression (Fig 5H). Gpr1p (plasma membrane G-protein coupled receptor), Gpa2p (G_α_), and Plc1p (phospholipase C) trigger filamentous growth by a pathway that is independent of fMAPK signaling (Fig 5A). *FRE*-driven expression was markedly abolished in *gpr1*Δ and *plc1*Δ, while *gpa2*Δ had no effect (Fig 5H).

Consistently, levels of phosphorylated Kss1p and Fus3p were almost abolished in cells carrying mutations in any genes of the pheromone pathway, with the exception of *STE2* (Fig 5I). Similarly, *gpr1*Δ and *plc1*Δ, but not mutations in other upstream regulators of filamentous growth also caused decreased phosphorylation (Fig 5I). Our data indicate that basal signaling through Kss1p in the S288C derived BY4741 strain is mediated by Gpr1p and the pheromone response pathway downstream of the pheromone receptor. Importantly, deletion of *ELM1* greatly elevated *FRE-lacZ* expression in *gpr1*Δ (Fig 5H), indicating that septin defects activate the pathway independent of Gpr1p.

We next asked if Fus3p’s kinase activity is required for regulating sadF pseudohyphal growth. The kinase-defective *fus3K42R* could partially suppress cell elongation and *FRE*-driven expression (S3 Fig). We conclude that Fus3p down-regulation of sadF pseudohyphal growth is only partially dependent on its kinase activity.

### The signaling pathway of sadF pseudohyphal growth

**Fig 6:**
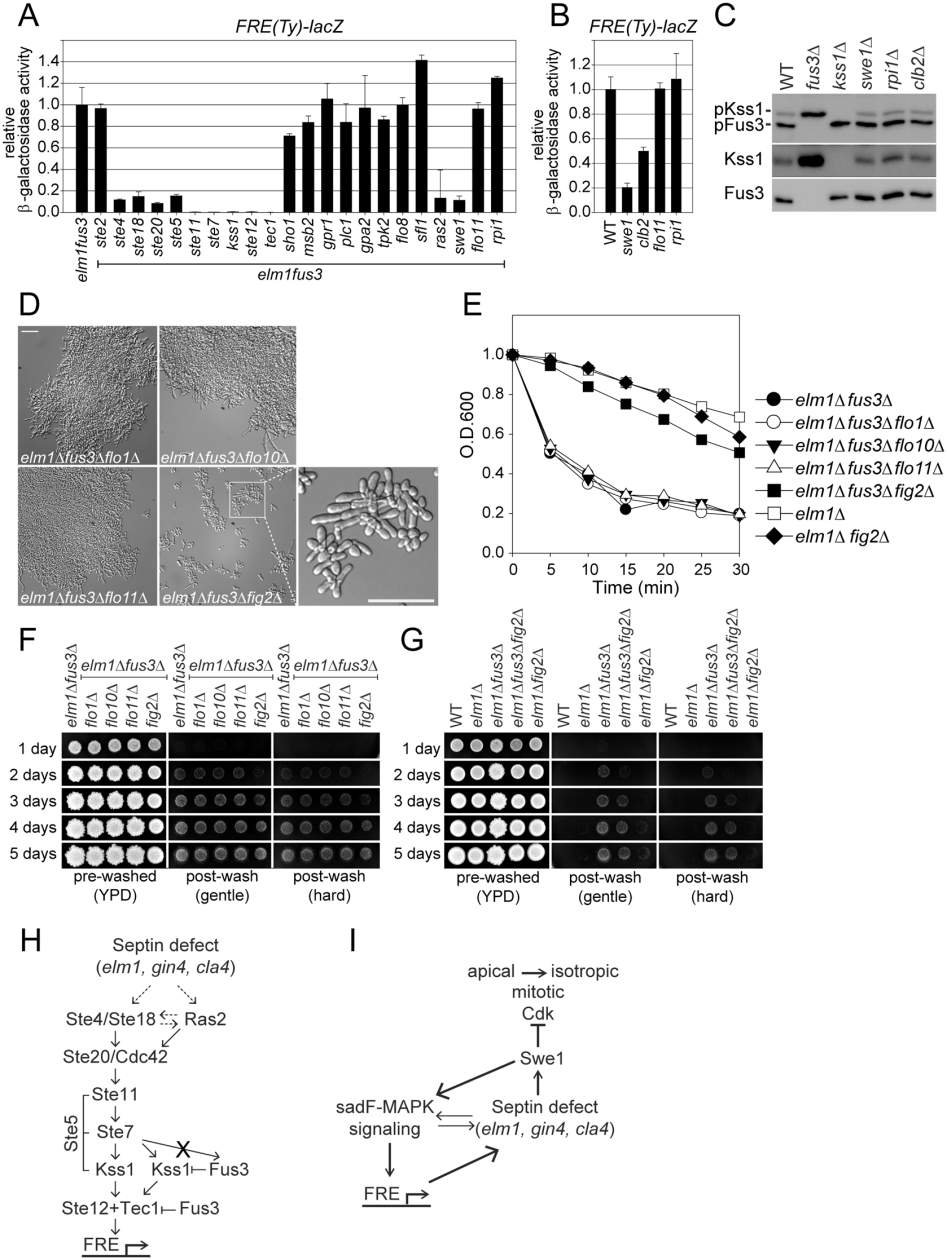
Identification of sadF pseudohyphal signaling pathway. All of strains were BY474-derivative haploids and grown to exponential phase in liquid YPD at 30°C unless stated otherwise. (A and B) Indicated strains (MY8092, 12948, 13122, 13125, 13145, 13281, 13313, 13315, 13317, 13319, 13378, 13380, 13382, 13384, 13413, 13417, 13500, 13502, 13504, 13506, 13508, 13531, 13582, 14331, 14332, 14333, 14334 and 14335) harboring *FRE(Ty)-lacZ* (MR6857) were used. β-galactosidase activity was determined as described in Fig 5C. Data are expressed relative to (A) *elm1*Δ *fus3*Δ or (B) WT. Data are mean ± standard deviation from three independent experiments. Note that (A) *elm1*Δ *fus3*Δ, *elm1*Δ *fus3*Δ *kss1*Δ, *elm1*Δ *fus3*Δ *tec1*Δ and (B) WT are identical to Fig 5C. (C) MY8092, 12886, 13144 and 13145 strains were used. Each protein was detected as described as Fig 5G. (D-G) Indicated strains (MY12158, 12948, 13413, 13901, 13903, 13907 and 14336) were used. (D) Bar, 30μm. (E) Flocculation assay was performed as described in Fig 1F. Data are representative of three independent experiments and expressed relative to WT. Note that *elm1*Δ and *elm1*Δ *fus3*Δare identical to Fig 1F. (F and G) Plate washing assay was performed as described in Fig 1G. (H and I) A model for sadF pseudohyphal signaling pathway. See the Discussion for details.

To identify the signaling pathway for sadF pseudohyphal growth, we screened the mutants for their effects on the induced level of *FRE-lacZ* in the *elm1*Δ *fus3*Δ mutant. Note that the *elm1*Δ *fus3*Δ induced level of *FRE-lacZ* expression is roughly 15-fold higher than the basal level (Fig 5C). As shown in Fig 6A, all the genes required for the basal level of *FRE*-driven expression are also essential for *elm1*Δ *fus3*Δ stimulated expression, with the exception of *GPR1* and *PLC1*. Deletion of either gene had no effect, indicating that Gpr1 and Plc1are not required for sadF pseudohyphal growth. As found for basal expression, the *msb2* and *sho1* mutants were essentially wild type (~70–80%) (Fig 6A). In contrast, deletion of *RAS2* largely abolished the induced level of *FRE*-driven expression, suggesting that defects in mitotic septin organization/function might stimulate sadF pseudohyphal signaling via Ras2p. Accordingly we investigated other components of the cAMP/PKA pathway, although BY4741 like other S288C strains carries a mutation in *FLO8*. As expected, *sfl1*Δ, *tpk2*Δ and *flo8*Δ did not cause any significant decrease in *FRE*-driven expression (Fig 6A). All triple mutants with significantly-reduced *FRE-lacZ* expression also failed to display the sadF pseudohyphal phenotype, consistent with the critical role for induced levels of Kss1p activation and Tec1p dependent transcription.

Given that *swe1*Δ suppresses sadF pseudohyphal growth, we examined its effect on *FRE*-driven transcription. Surprisingly, loss of Swe1p also strongly suppressed *FRE*-driven transcription in *elm1*Δ *fus3*Δ (Fig 6A). The *swe1*Δ also reduced basal expression of *FRE-lacZ* (Fig 6B), but it had little effect on basal Kss1p phosphorylation (Fig 6C), suggesting that Swe1p acts downstream of Kss1p to regulate *FRE*-driven expression. Because Swe1p negatively regulates the mitotic Cdk, the reduction in *FRE*-driven expression by deletion of *SWE1* might be due to the increased Cdk activity. If so, then *FRE*-driven expression should be upregulated in a strain lacking Clb2p, the major mitotic cyclin. However, *clb2*Δ did not stimulate *FRE*-reporter expression; instead, cells exhibited a 2-fold reduction (Fig 6C). These results suggest that Swe1p’s function in sadF pseudohyphal signaling is not solely dependent on reduction of mitotic Cdk activity.

The classic forms of filamentous growth require Flo11p, a cell surface flocculin, to promote filamentation and agar invasion. We therefore examined whether sadF pseudohyphae are Flo11p-dependent. Deletion of *FLO11* had no effect on sadF pseudohyphal signaling (Fig 6A), basal *FRE*-driven expression (Fig 6B), filamentation (Fig 6D), aggregation (Fig 6E) or agar invasion of *elm1*Δ *fus3*Δ cells (Fig 6F). Recently it was shown that S288C strains bypass fMAPK signaling for Flo11p induction; instead, Rpi1p regulates transcription of *FLO11* [30]. The *rpi1*Δ had no effect on the sadF pseudohyphal phenotype or signaling (Fig 6A and 6B) again showing that sadF pseudohyphal growth is independent of Flo11p and the cAMP/PKA pathway.

Budding yeast has several cell wall proteins related to the adhesins of pathogenic fungi including Flo1p, Flo10p, Flo11p, and Fig2p. Flo1p and Flo10p promote cell-cell adhesion to form multicellular clumps, leading to flocculation [31]. Fig2p is induced during mating and implicated in cell-cell adherence for cell fusion [32]. As shown in Fig 6D–6F, *flo1*Δ and *flo10*Δ had no effect on the morphology, clumping or invasiveness of sadF pseudohyphal cells. In contrast, *fig2*Δ partially suppressed sadF pseudohyphal growth, showing reduced cell clump size, reduced clumping, and reduced agar invasion of the *elm1*Δ *fus3*Δ strain (Fig 6D–6G). Thus sadF pseudohyphal growth also differs from the classical forms of filamentation in requiring a different adhesin to promote multicellular growth.

## Discussion

In this paper we report the discovery of a stable constitutive pseudohyphal growth state for the budding yeast, *Saccharomyces cerevisiae*. The constitutive pseudohyphal growth, referred to as sadF growth, is triggered by two regulatory inputs: perturbation in the assembly of the septin ring required for cytokinesis and relief of the negative regulation of Kss1p by the mating pathway MAP kinase, Fus3p. As for classical filamentous growth, sadF growth is characterized by hyperpolarized cell growth, a unipolar budding pattern and increased cell adhesion, leading to growth as large clumps of branched filaments. Although sadF pseudohyphal growth shares some characteristics with classical diploid pseudohyphal growth, it differs in significant ways, including the conditions in which it occurs (liquid rich media versus solid starvation media), the overall genetic requirements (haploid S288C background versus diploid ∑), the regulatory pathway (dependent on Ste5p, but independent of the cAMP/PKA pathway and Flo11p), and the degree of hyperpolarized growth. Besides nitrogen starvation, various environmental stimuli such as alcohol and slowed DNA synthesis have also been revealed to promote filamentation [33, 34]; short-chain alcohols were able to stimulate filamentous growth even in liquid media with properties similar to classical pseudohyphal growth. However, these filamentous growths were not induced in strains of the S288C lineage [33, 34].

The sadF pseudohyphal cells achieve a degree of polarized growth that is significantly more extreme than that previously reported for diploid filamentous growth (mean axial ratio of 4.3, versus 3.4; [2]). However, the distribution of sadF pseudohyphal cell lengths was highly variable, with some cells superficially resembling the hyphae of true filamentous fungi. Like filamentous fungi, sadF pseudohyphal yeast contain a Spitzenkörper-like structure at the tips of the growing cells (Fig 3D). Comprised of secretory and endocytic vesicles, the Spitzenkörper is thought to be a staging area for secreted material that will form the new cell surface. During budding, *S*. *cerevisiae* growth is isotropic over the bud surface, with no evidence of a Spitzenkörper. Although Spitzenkörper have not been reported in *S*. *cerevisiae* pseudohyphal growth, in the related human pathogen, *Candida albicans*, which can grow both as true hyphae and as pseudohyphae, Spitzenkörper are observed only in true hyphae [35]. Interestingly, a Spitzenkörper-like structure is seen in budding yeast during the response to pheromone ([36], Bagamery and Rose, submitted), indicating that budding yeast have the machinery for Spitzenkörper formation. Moreover, a Spitzenkörper-like structure is observed in budding yeast, early in the cell cycle, prior to the “apical to isotropic” switch (Bagamery and Rose, submitted). Thus it seems likely that the sadF hyper-polarized growth reflects the maintenance of a growth pattern that is normally restricted to mating and/or the early phases of the cell cycle.

Septin assembly defects appear to induce pseudohyphal growth through two different signaling pathways (Fig 6H and 6I). First, septin defects activate a MAP kinase pathway (sadF-MAPK) comprising most components of the pheromone response pathway, including ones that are not shared with the pseudo-hyphal signaling pathway (Ste4p/Ste18p and Ste5p). It is not known how the sadF-MAPK signaling pathway is activated by septin defects, although we note the presence of increased levels of Ste5-GFP at cortical puncta and the bud neck in strains undergoing sadF pseudohyphal growth (S4 Fig). Septin assembly must be normally monitored by the sadF-MAPK pathway; mutations affecting septins caused up to 3 fold higher Kss1p phosphorylation and Tec1p-dependent *FRE*-reporter expression. Under normal conditions, the expression of the filamentation response genes is ameliorated by Fus3p. In the absence of Fus3p, filamentation response gene expression is greatly enhanced; the expression of which leads to further suppression of septin assembly and the pronounced changes in cell morphology characteristic of pseudohyphal growth.

Septin assembly defects is also monitored by Swe1p, whose activation leads to cell cycle arrest prior to the switch from apical to isotropic growth [20]. A role for Swe1p-dependent cell-cycle arrest in sadF pseudohyphal growth is supported by the finding that loss of Mih1p (which antagonizes Swe1p activity) leads to cell elongation in a *fus3*Δ mutant (Fig 4F). At the same time, Swe1p is also required for high level sadF-MAPK signaling. Surprisingly, Swe1p does not appear to be required upstream in the signaling pathway; Swe1p is not required for Kss1p phosphorylation. To explain this finding, we propose that activation of the Swe1p-dependent septin assembly checkpoint blocks cell cycle progression at the precise stage during which septin assembly is actively being monitored by the sadF-MAPK pathway. Because high-level expression of the filamentation response genes appears to exacerbate septin assembly defects (S2 Fig), cells become locked in a state in which both Swe1p and sadF-MAPK signaling remain high, essentially creating a positive feedback loop (Fig 6H). Because cells remain blocked in the cell cycle prior to the switch from apical to isotropic growth, they continue to grow in a highly polarized, hyphal-like fashion. The cell cycle block is not permanent as cells eventually succeed at completing cytokinesis, albeit after most have grown to a length that is at least 4 to 5 times longer than wild-type.

We presume that other cellular functions associated with the filamentation response genes contribute to the unique cell morphology of sadF pseudohyphal growth. For example, loss of Fus3p is sufficient to switch about 30% of cells from the haploid axial budding pattern to the bipolar budding pattern (Fig 2), rising to 90% when coupled to *elm1*Δ. Similarly, the Fig2p adhesin is required for the full level of cell adhesion. Although *FIG2* has not been reported to be a filamentation response gene, overexpression causes increased filamentation and the promoter region binds Phd1p, a transcription factor that enhances filamentous growth. Moreover, in *Ashbya gossypii FIG2* expression was abolished in a *tec1* mutant [37]. Thus the sadF pseudohyphal growth state can be viewed as the product of synergism between the set of genes which regulate aspects of cell morphology and genes which regulate progression through the cell cycle.

Aside from notable differences in the regulatory mechanism, sadF pseudohyphal growth is remarkable because it is a stable growth state that is independent of the external environmental cues that trigger classical pseudohyphal growth. The various growth states of budding yeast can be thought of as being dominated by the activity of specific master regulatory protein kinases. For example, mitosis is dominated by the activity of the CDK, Cdc28p. Mating is dominated by the activity of the MAPK, Fus3p. Meiosis is dominated by Ime1p. Each growth state activates mechanisms to suppress the activity of alternate growth phase kinases. For example, phosphorylation of Ste5p by Cdc28p blocks activation of Fus3p prior to G2 [38]. During mating, activation of Far1p by Fus3p blocks activation of Cdc28p to prevent reentry into mitosis. Similarly, sadF pseudohyphal growth can be thought of as being dominated by Swe1p and Kss1p, which synergistically activate genes responsible for the alternate growth state and block Cdc28p-dependent mitotic progression.

Much recent interest has focused on the mechanism by which unicellular organisms transitioned to multicellular patterns of growth. Using different selection schemes two different laboratory groups have evolved yeast strains into forms that show a striking degree of multicellularity [39,40]. In both cases cells showed dramatic increases in the level of adhesion, although neither showed significant changes in cell morphology. The relative ease of the transition for budding yeast has suggested that the evolution of multicellularity might be generally easy for unicellular eukaryotes under proper selective pressure. Our results show that loss of function mutations in just two genes can also induce a high degree of multicellularity in budding yeast. Thus this organism may already contain the genomic complexity required for robust multicellular growth.

## Materials and Methods

### Yeast strains, plasmids, growth conditions and general methods

All yeast culture and genetic techniques were performed as described by [41]. Yeast strains and plasmids are listed in S1 Table. All yeast strains are haploid congenic to the S288C genetic background and were grown in liquid culture of rich YPD (yeast extract/peptone/dextrose) media unless indicated otherwise. The gene disruption and GFP tagging of *HTB1* were performed by PCR-based methods [42]. To determine cell-cell adhesion quantitatively, a flocculation assay was performed as described previously [43]. Briefly, exponentially growing cells in liquid YPD media were thoroughly mixed and the absorbance (A600) was determined immediately (t = 0) with a spectrophotometer. Optical density was read at 5 min intervals for 30 min without agitation and normalized by those of t = 0. β-galactosidase assays were performed with extracts from exponentially growing cells in synthetic minimal medium containing 2% glucose as described previously [29].

### Protein analysis

Proteins were prepared by TCA precipitation as described previously [41] and probed with polyclonal anti-Kss1p (Santa Cruz), polyclonal anti-Fus3p (Santa Cruz), monoclonal anti-phosphor-p44/42 MAPK (Cell signaling), and polyclonal Kar2p (Rose Lab, Princeton University). Band intensity was quantified using G:BOX imaging system (Syngene).

### Microscopy and cell imaging

Images for colony morphology were acquired with G:BOX imaging system (Syngene). Florescence microscopy was performed essentially as described previously [44]. Images were acquired on a DeltaVision Microscopy System (Applied Precision, LLC) using an inverted microscope (TE200; Nikon), a charge-coupled device camera (CoolSNAP HQ; Roper Scientific) and either a 40× objective with a 0.75 NA or a 20× with a 0.50 NA (Nikon). Figures were prepared for publication using Adobe Photoshop and Adobe Illustrator. No further manipulations other than adjustments in brightness and contrast were made. To determine DNA contents, cells were grown to exponential phase, fixed with 70% ethanol, and treated with RNAase. The cells were then stained with propidium iodide overnight at 4°C and fluorescent intensity was quantified with CellProfiler image analysis software [45]. For time-lapse experiments, cells were grown to early exponential phase in liquid YPD medium and were placed on YPD agarose pads. 10 z sections spaced 1 μm apart without binning were acquired every 2 min. All images were projected and processed with the softWoRx program (Applied Precision, LLC).

## Supporting Information

S1 Fig Cell adhesion and invasive growth of *gin4*Δ *fus3*Δ and *cla4*Δ *fus3*Δ mutants. All of strains were BY474-derivative haploids and grown to exponential phase in liquid YPD at 30°C. MY8091, 12156, 12871, 12886, 12941, 12944, and 12990 strains were used. (A) Flocculation assay was performed as described in Fig 1F. Data are representative of three independent experiments and expressed relative to wild type. Note that wild type and *fus3*Δ are identical to Fig 1F. (B) Plate washing assay was performed as described in Fig 1G.
(TIF)

S2 Fig Genetic interaction of *fus3*Δ mutation with septin mutants. All of strains were BY474-derivative haploids. MY8092, 12886, 12969, 12970, 14050, 14054, 14056, 14058, 14132, 14134, 14136 and 14139 strains were used. The temperature-sensitive *cdc3-3*, *cdc11-6* and *cdc12-6* mutants were constructed in BY4741. (A) Cells were serially diluted on YPD and incubated at 23°C for 2 days, 30°C for 1 day, or 37°C for 1 day. (B) Cells grown to exponential phase in YPD liquid at 23°C were transferred to 30°C for 12 hr. Bar, 10 μm. To further establish the relationship between Fus3p and septin perturbation in pseudohyphal growth, we examined the effect of *fus3*Δ on the growth and morphology of septin mutants. Neither Cdc10p nor Shs1p are essential in BY4741 and their absence resulted only in a slightly elongated bud phenotype that was not sensitive to elevated temperature. In contrast, Cdc3p, Cdc11p and Cdc12p are essential proteins and temperature-sensitive mutants displayed elongated cell morphology that became significantly more severe at elevated temperatures, becoming inviable at 37°C. The different septin mutations exhibited a variety of interactions with *fus3*Δ. Four of the five mutations (*cdc3-3*, *cdc10*Δ, *cdc12-6*, and *shs1*Δ) showed strong synthetic growth defects with *fus3*Δ, such that the growth of the double mutants was greatly reduced relative to either single mutant at otherwise permissive temperatures (S2A Fig). Two of the double mutants, *cdc3-3 fus3*Δ and *cdc12-6 fus3*Δ, displayed more severe morphologies with extremely elongated buds at the intermediate temperature of 30°C (S2B Fig). Although *shs1*Δ *fus3*Δ double mutant did not show elongated buds, the cells aggregated into clumps that were not dispersed by sonication (S2B Fig). In contrast to the other septin mutants, *fus3*Δ did not cause an obvious synthetic growth defect in combination with *cdc11-6* (S2A Fig) and the effect of *fus3*Δ on *cdc11-6* morphology was less severe compared to *cdc3-3* and *cdc12-6* (S2B Fig). Taken together, these interactions suggest that the various septin mutations perturb septation in a variety of ways leading to differential genetic interactions with *fus3*Δ.
(TIF)

S3 Fig Fus3p kinase activity in sadF pseudohyphal growth. *elm1*Δ *fus3*Δ (MY12948) harboring kinase-defective *pCEN-fus3K42R* (MR6763) were grown in liquid SC contacting 2% glucose and photographed (left). *elm1*Δ*fus3*Δ (MY12948) harboring either *pCEN-FUS3* (MR5048) or *pCEN-fus3K42R* (MR6763) were transformed with *FRE(Ty)-lacZ* (MR6857) and β-galactosidase activity was determined as described in Materials and Methods. Bar, 10 μm.
(TIF)

S4 Fig Recruitment of Ste5p-3GFP at the plasma membrane in *elm1*Δ *fus3*Δ and *gin4*Δ *fus3*Δ mutants. Indicated cells (MY13378, 13394 and 14322) harboring pCEN-STE5-3GFP (pMR6725) were grown to exponential phase in liquid SC media containing glucose at 30°C. GFP fluorescence was observed in living cells. Bar, 10 μm.
(TIF)

S1 Table Yeast strains and plasmids used in this study. Unless indicated otherwise, all strains were constructed in this study.
(DOCX)
