# Supplementary figures and images for: Stable Pseudohyphal Growth in Budding Yeast Induced by Synergism between Septin Defects and Altered MAP-kinase Signaling
Source: PLoS Genet. 2015 Dec 7;11(12):e1005684. doi: 10.1371/journal.pgen.1005684 (PMC4671653; doi:10.1371/journal.pgen.1005684)

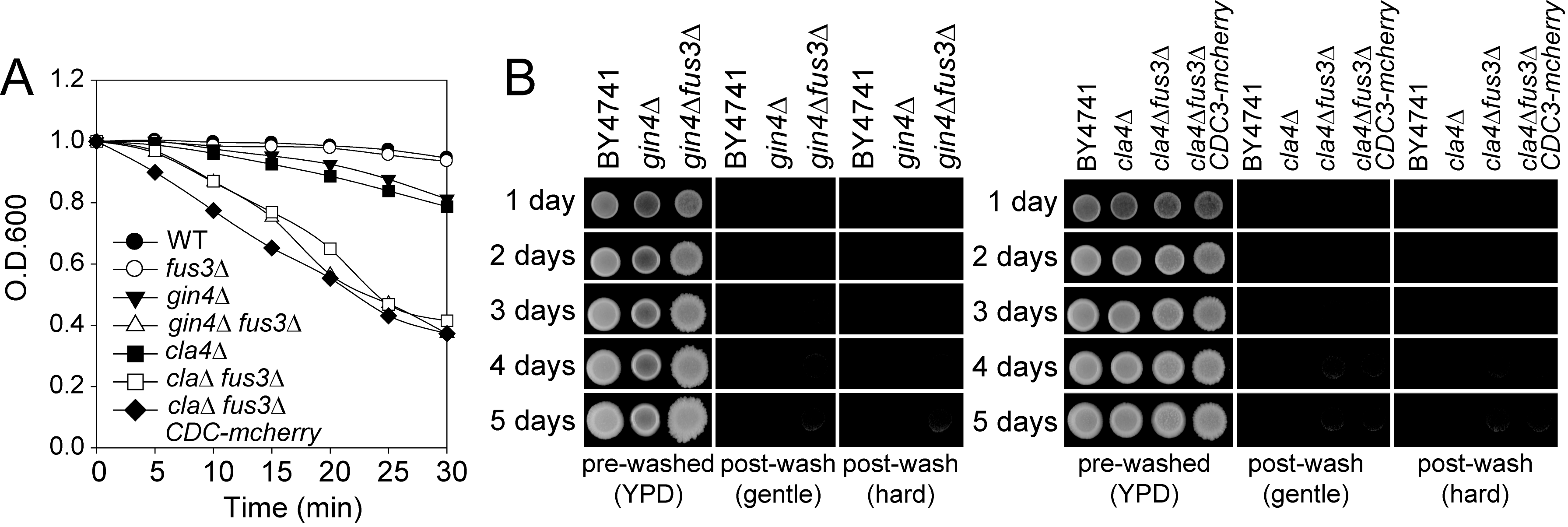

Supplement: S1 Fig — All of strains were BY474-derivative haploids and grown to exponential phase in liquid YPD at 30°C. MY8091, 12156, 12871, 12886, 12941, 12944, and 12990 strains were used. (A) Flocculation assay was performed as described in Fig 1F. Data are representative of three independent experiments and expressed relative to wild type. Note that wild type and fus3Δ are identical to Fig 1F. (B) Plate washing assay was performed as described in Fig 1G. (TIF) [file pgen.1005684.s001.tif]

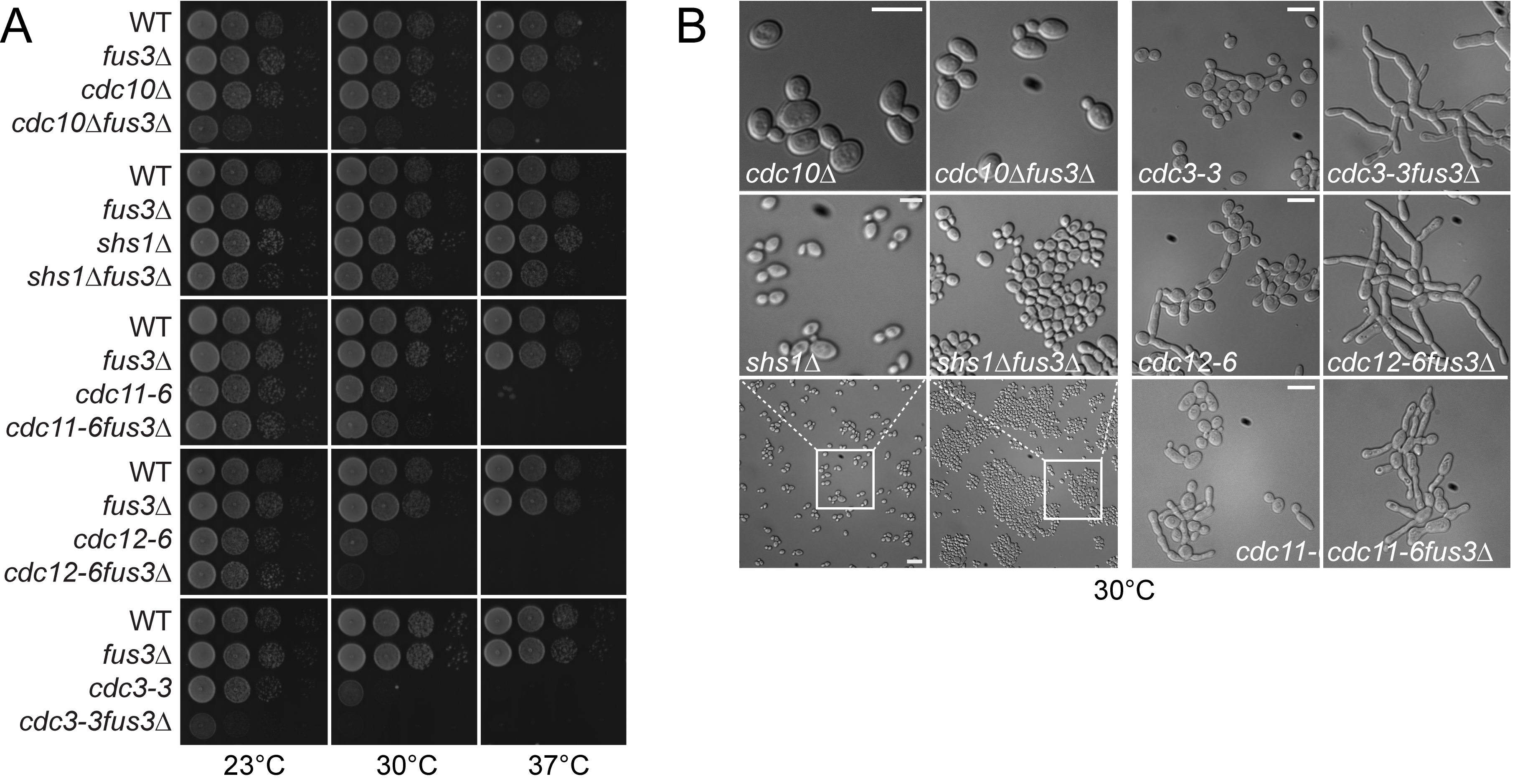

Supplement: S2 Fig — All of strains were BY474-derivative haploids. MY8092, 12886, 12969, 12970, 14050, 14054, 14056, 14058, 14132, 14134, 14136 and 14139 strains were used. The temperature-sensitive cdc3-3, cdc11-6 and cdc12-6 mutants were constructed in BY4741. (A) Cells were serially diluted on YPD and incubated at 23°C for 2 days, 30°C for 1 day, or 37°C for 1 day. (B) Cells grown to exponential phase in YPD liquid at 23°C were transferred to 30°C for 12 hr. Bar, 10 μm. To further establish the relationship between Fus3p and septin perturbation in pseudohyphal growth, we examined the effect of fus3Δ on the growth and morphology of septin mutants. Neither Cdc10p nor Shs1p are essential in BY4741 and their absence resulted only in a slightly elongated bud phenotype that was not sensitive to elevated temperature. In contrast, Cdc3p, Cdc11p and Cdc12p are essential proteins and temperature-sensitive mutants displayed elongated cell morphology that became significantly more severe at elevated temperatures, becoming inviable at 37°C. The different septin mutations exhibited a variety of interactions with fus3Δ. Four of the five mutations (cdc3-3, cdc10Δ, cdc12-6, and shs1Δ) showed strong synthetic growth defects with fus3Δ, such that the growth of the double mutants was greatly reduced relative to either single mutant at otherwise permissive temperatures (S2A Fig). Two of the double mutants, cdc3-3 fus3Δ and cdc12-6 fus3Δ, displayed more severe morphologies with extremely elongated buds at the intermediate temperature of 30°C (S2B Fig). Although shs1Δ fus3Δ double mutant did not show elongated buds, the cells aggregated into clumps that were not dispersed by sonication (S2B Fig). In contrast to the other septin mutants, fus3Δ did not cause an obvious synthetic growth defect in combination with cdc11-6 (S2A Fig) and the effect of fus3Δ on cdc11-6 morphology was less severe compared to cdc3-3 and cdc12-6 (S2B Fig). Taken together, these interactions suggest that the various septin mutations [file pgen.1005684.s002.tif]

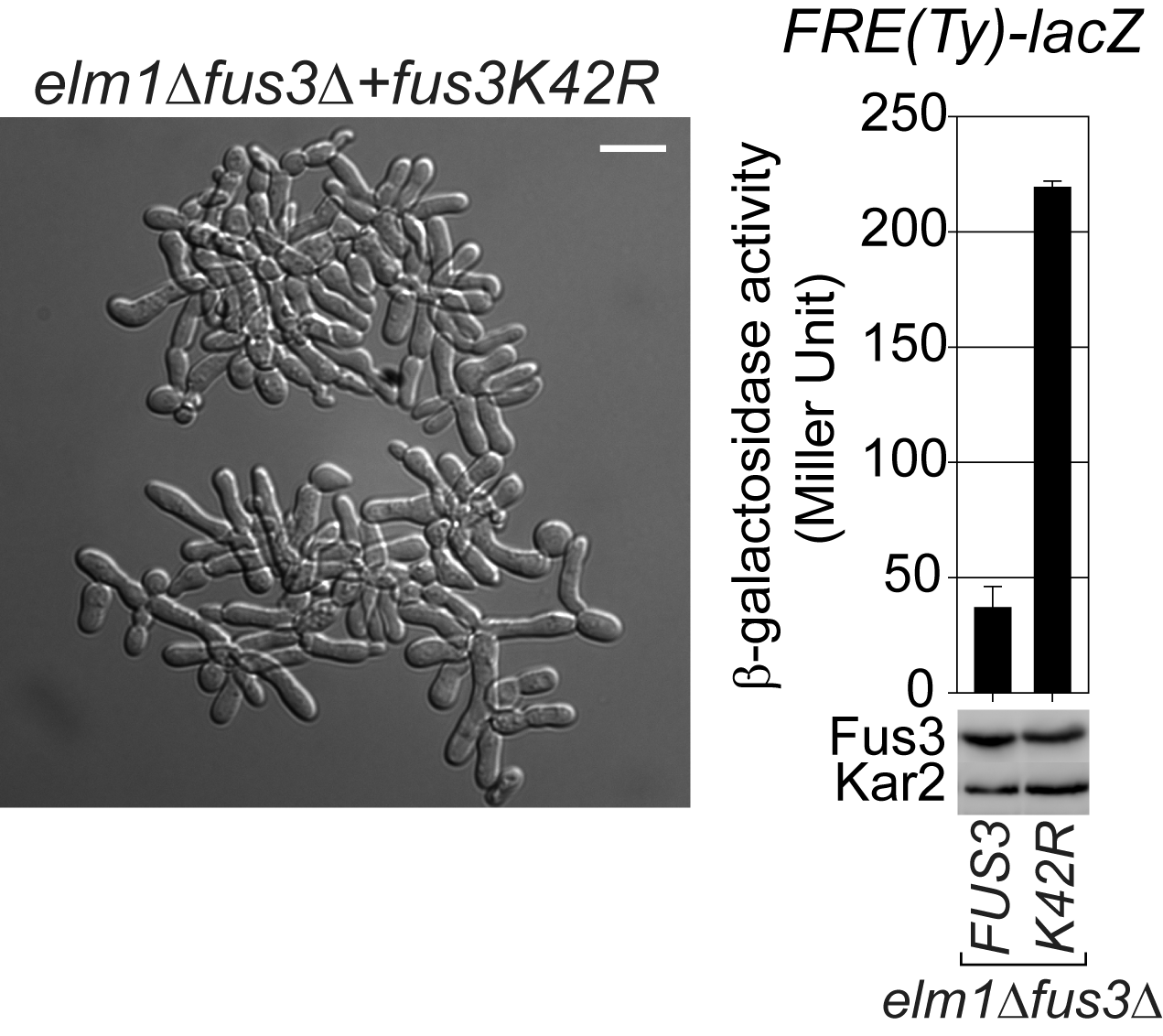

Supplement: S3 Fig — elm1Δ fus3Δ (MY12948) harboring kinase-defective pCEN-fus3K42R (MR6763) were grown in liquid SC contacting 2% glucose and photographed (left). elm1Δfus3Δ (MY12948) harboring either pCEN-FUS3 (MR5048) or pCEN-fus3K42R (MR6763) were transformed with FRE(Ty)-lacZ (MR6857) and β-galactosidase activity was determined as described in Materials and Methods. Bar, 10 μm. (TIF) [file pgen.1005684.s003.tif]

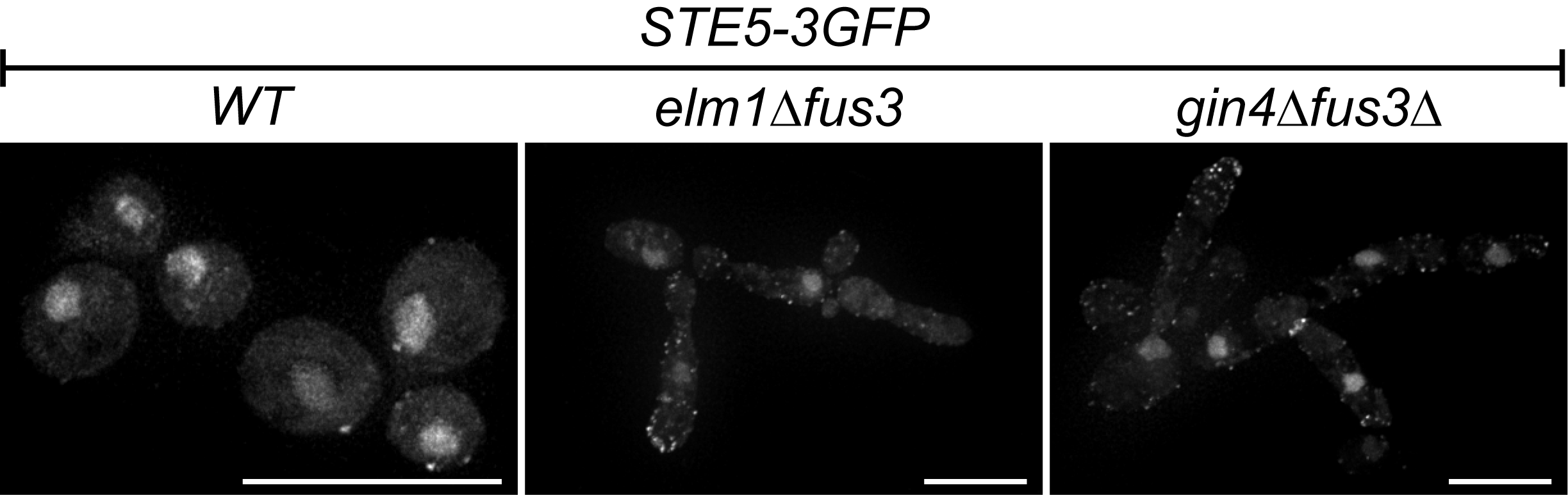

Supplement: S4 Fig — Indicated cells (MY13378, 13394 and 14322) harboring pCEN-STE5-3GFP (pMR6725) were grown to exponential phase in liquid SC media containing glucose at 30°C. GFP fluorescence was observed in living cells. Bar, 10 μm. (TIF) [file pgen.1005684.s004.tif]
