# Supplementary material for: Stable Pseudohyphal Growth in Budding Yeast Induced by Synergism between Septin Defects and Altered MAP-kinase Signaling
Source: PLoS Genet. 2015 Dec 7;11(12):e1005684. doi: 10.1371/journal.pgen.1005684 (PMC4671653; doi:10.1371/journal.pgen.1005684)
Supplement: S1 Table — Unless indicated otherwise, all strains were constructed in this study. (DOCX) [file pgen.1005684.s005.docx]

**Table S1: Strains and plasmids used in this study**

| Strain | Genotype | Source or reference* |
| --- | --- | --- |
| Isogenic to S288C | | |
| MY8092 | *MAT***a** *ura3-*Δ*0 his3-*Δ*200 leu2-*Δ*0 met15-*Δ*0* | BY4741 |
| MY12156 | BY4741 *except cla4*Δ::*kanMX4* | Research Genetics |
| MY12158 | BY4741 *except elm1*Δ::*kanMX4* | Research Genetics |
| MY12871 | BY4741 *except cla4*Δ::*natMX6 fus3*Δ::*HIS3 Cdc3-mcherry:kanMX6* | |
| MY12886 | BY4741 *except fus3*Δ::*kanMX4* | Research Genetics |
| MY12941 | BY4741 *except gin4*Δ::*kanMX4* | Research Genetics |
| MY12944 | BY4741 *except gin4*Δ::*kanMX4* Δ*fus3*::*natMX6* |  |
| MY12948 | BY4741 *except elm1*Δ::*kanMX4* Δ*fus3*::*natMX6* |  |
| MY12960 | BY4741 *except mih1*Δ::*kanMX4* | Research Genetics |
| MY12961 | BY4741 *except mih1*Δ::*kanMX4* Δ*fus3*::*natMX6* |  |
| MY12969 | BY4741 *except shs1*Δ::*kanMX4* | Research Genetics |
| MY12970 | BY4741 *except shs1*Δ::*kanMX4* Δ*fus3*::*natMX6* |  |
| MY12990 | BY4741 *except cla4*Δ::*natMX6* Δ*fus3*::*HIS3* |  |
| MY13049 | BY4741 *except tec1*Δ::*kanMX4* | Research Genetics |
| MY13122 | BY4741 *except elm1*Δ::*kanMX4 fus3*Δ::*natMX6 kss1*Δ::*hphMX4* |  |
| MY13125 | BY4741 *except elm1*Δ::*kanMX4 fus3*Δ::*natMX6 swe1*Δ::*hphMX4* |  |
| MY13144 | BY4741 *except kss1*Δ::*kanMX4* | Research Genetics |
| MY13145 | BY4741 *except swe1*Δ::*kanMX4* | Research Genetics |
| MY13161 | BY4741 *except elm1*Δ::*kanMX4 Cdc3-mcherry:his5^+^* |  |
| MY13264 | BY4741 *except elm1*Δ::*kanMX4* Δ*fus3*::*natMX6 Cdc3-mcherry:his5^+^* |  |
| MY13281 | BY4741 *except elm1*Δ::*kanMX4 fus3*Δ::*natMX6 tec1*Δ::*hphMX4* |  |
| MY13289 | BY4741 *except fus3*Δ::*kanMX4 kss1*Δ::*hphMX4* |  |
| MY13313 | BY4741 *except elm1*Δ::*kanMX4 fus3*Δ::*natMX6 ste7*Δ::*hphMX4* |  |
| MY13315 | BY4741 *except elm1*Δ::*kanMX4 fus3*Δ::*natMX6 ste11*Δ::*hphMX4* |  |
| MY13317 | BY4741 *except elm1*Δ::*kanMX4 fus3*Δ::*natMX6 ste20*Δ::*hphMX4* |  |
| MY13319 | BY4741 *except elm1*Δ::*kanMX4 fus3*Δ::*natMX6 flo8*Δ::*hphMX4* |  |
| MY13378 | BY4741 *except elm1*Δ::*kanMX4 fus3*Δ::*natMX6 ste5*Δ::*hphMX4* |  |
| MY13380 | BY4741 *except elm1*Δ::*kanMX4 fus3*Δ::*natMX6 ras2*Δ::*hphMX4* |  |
| MY13382 | BY4741 *except elm1*Δ::*kanMX4 fus3*Δ::*natMX6 sfl1*Δ::*hphMX4* |  |
| MY13384 | BY4741 *except elm1*Δ::*kanMX4 fus3*Δ::*natMX6 tpk2*Δ::*hphMX4* |  |
| MY13394 | BY4741 *except gin4*Δ::*kanMX4 fus3*Δ::*natMX6 tpk2*Δ::*hphMX4* |  |
| MY13411 | *MAT***a***/*α *ura3/ura3 trp1*Δ*1/TRP1 his3-*Δ*200/ his3-*Δ*200 LEU2/ leu2-*Δ*1* |  |
| MY13413 | BY4741 *except elm1*Δ::*kanMX4 fus3*Δ::*natMX6 flo11*Δ::*hphMX4* |  |
| MY13417 | BY4741 *except elm1*Δ::*kanMX4 fus3*Δ::*natMX6 ste12*Δ::*hphMX4* |  |
| MY13484 | BY4741 *except elm1*Δ::*kanMX4 gpr1*Δ::*hphMX4* |  |
| MY13500 | BY4741 *except elm1*Δ::*kanMX4 fus3*Δ::*natMX6 ste4*Δ::*hphMX4* |  |
| MY13502 | BY4741 *except elm1*Δ::*kanMX4 fus3*Δ::*natMX6 msb2*Δ::*hphMX4* |  |
| MY13504 | BY4741 *except elm1*Δ::*kanMX4 fus3*Δ::*natMX6 gpr1*Δ::*hphMX4* |  |
| MY13506 | BY4741 *except elm1*Δ::*kanMX4 fus3*Δ::*natMX6 ste2*Δ::*hphMX4* |  |
| MY13508 | BY4741 *except elm1*Δ::*kanMX4 fus3*Δ::*natMX6 sho1*Δ::*hphMX4* |  |
| MY13523 | BY4741 *except ste12*Δ::*hphMX4* |  |
| MY13525 | BY4741 *except ste18*Δ::*hphMX4* |  |
| MY13531 | BY4741 *except elm1*Δ::*kanMX4 fus3*Δ::*natMX6 ste18*Δ::*hphMX4* |  |
| MY13582 | BY4741 *except elm1*Δ::*kanMX4 fus3*Δ::*natMX6 rpi1*Δ::*hphMX4* |  |
| MY13817 | BY4741 *except Htb1-GFP*::*his5^+^* |  |
| MY13820 | BY4741 *except elm1*Δ::*kanMX4 fus3*Δ::*natMX6 Htb1-GFP*::*his5^+^* |  |
| MY13826 | BY4741 *except snf1*Δ::*kanMX4* | Research Genetics |
| MY13827 | BY4741 *except snf1*Δ::*kanMX4 fus3*Δ::*natMX6* |  |
| MY13829 | BY4741 *except kin4*Δ::*kanMX4* | Research Genetics |
| MY13830 | BY4741 *except kin4*Δ::*kanMX4 fus3*Δ::*natMX6* |  |
| MY13832 | BY4741 *except hsl1*Δ::*kanMX4* | Research Genetics |
| MY13833 | BY4741 *except hsl1*Δ::*kanMX4 fus3*Δ::*natMX6* |  |
| MY13901 | BY4741 *except elm1*Δ::*natMX4 fus3*Δ::*kanMX4 flo1*Δ::*hphMX4* |  |
| MY13903 | BY4741 *except elm1*Δ::*natMX4 fus3*Δ::*kanMX4 flo10*Δ::*hphMX4* |  |
| MY13907 | BY4741 *except elm1*Δ::*natMX4 fus3*Δ::*kanMX4 fig2*Δ::*hphMX4* |  |
| MY14050 | BY4741 *except cdc11*::*cdc11-6*::*hphMX4* |  |
| MY14054 | BY4741 *except cdc12*::*cdc12-6*::*hphMX4* |  |
| MY14056 | BY4741 *except fus3*Δ::*kanMX4 cdc12*::*cdc12-6*::*hphMX4* |  |
| MY14058 | BY4741 *except cdc10*Δ::*kanMX4* | Research Genetics |
| MY14132 | BY4741 *except fus3*Δ::*natMX6 cdc11*::*cdc11-6*::*hphMX4* |  |
| MY14134 | BY4741 *except cdc3*::*cdc3-3*::*hphMX4* |  |
| MY14136 | BY4741 *except fus3*Δ::*kanMX4 cdc3*::*cdc3-3*::*hphMX4* |  |
| MY14139 | BY4741 *except cdc10*::*kanMX4 fus3*Δ::*natMX4* |  |
| MY14319 | BY4741 *except ste2*Δ::*kanMX4* | Research Genetics |
| MY14320 | BY4741 *except ste4*Δ::*kanMX4* | Research Genetics |
| MY14321 | BY4741 *except ste20*Δ::*kanMX4* | Research Genetics |
| MY14322 | BY4741 *except ste5*Δ::*kanMX4* | Research Genetics |
| MY14323 | BY4741 *except ste11*Δ::*kanMX4* | Research Genetics |
| MY14324 | BY4741 *except ste7*Δ::*kanMX4* | Research Genetics |
| MY14325 | BY4741 *except gpr1*Δ::*kanMX4* | Research Genetics |
| MY14326 | BY4741 *except gpa2*Δ::*kanMX4* | Research Genetics |
| MY14327 | BY4741 *except plc1*Δ::*kanMX4* | Research Genetics |
| MY14328 | BY4741 *except ras2*Δ::*kanMX4* | Research Genetics |
| MY14329 | BY4741 *except msb2*Δ::*kanMX4* | Research Genetics |
| MY14330 | BY4741 *except sho1*Δ::*kanMX4* | Research Genetics |
| MY14331 | BY4741 *except elm1*Δ::*kanMX4 fus3*Δ::*kanMX4 plc1*Δ::*kanMX4* |  |
| MY14332 | BY4741 *except elm1*Δ::*kanMX4 fus3*Δ::*kanMX4 gpa2*Δ::*kanMX4* |  |
| MY14333 | BY4741 *except clb2*Δ::*kanMX4* | Research Genetics |
| MY14334 | BY4741 *except flo11*Δ::*kanMX4* | Research Genetics |
| MY14335 | BY4741 *except rpi1*Δ::*kanMX4* | Research Genetics |
| MY14336 | BY4741 *except elm1*Δ::*kanMX4 fig2*Δ::*kanMX4* |  |
| Isogenic to Sigma1278b | | |
| MY13465 | *MAT***a** *his3*Δ::*hisG leu2*Δ::*hisG trp1*Δ::*hisG ura3-52* | J. Thorner |
| Plasmid | *Description* | Source or reference* |
| pMR0727 | *CEN URA3 fus1-lacZ* | G. R. Fink |
| pMR1865 | *HIS3 CEN6 ARS4 amp-r lacZ' f1 T3-promoter T7-promoter* | pRS413 |
| pMR5048 | *FLAG-FUS3 CEN HIS3* | Dina Matheos |
| pMR6763 | *FLAG-fus3 K42R CEN HIS3* |  |
| pMR6725 | *CEN URA3 STE5-3GFP* | J. Nickels |
| pMR6857 | *YEpU-FTyZ* | J. Thorner |

*All of cells and plasmids without source were constructed in this study.
